# Supplementary material for: Detailed characterisation of the trypanosome nuclear pore architecture reveals conserved asymmetrical functional hubs that drive mRNA export
Source: PLoS Biol. 2025 Feb 3;23(2):e3003024. doi: 10.1371/journal.pbio.3003024 (PMC11825100; doi:10.1371/journal.pbio.3003024)
Supplement: S1 Fig — (PDF) [file pbio.3003024.s001.pdf]

Figure S1 (page 1/2)

A principle of ProExM

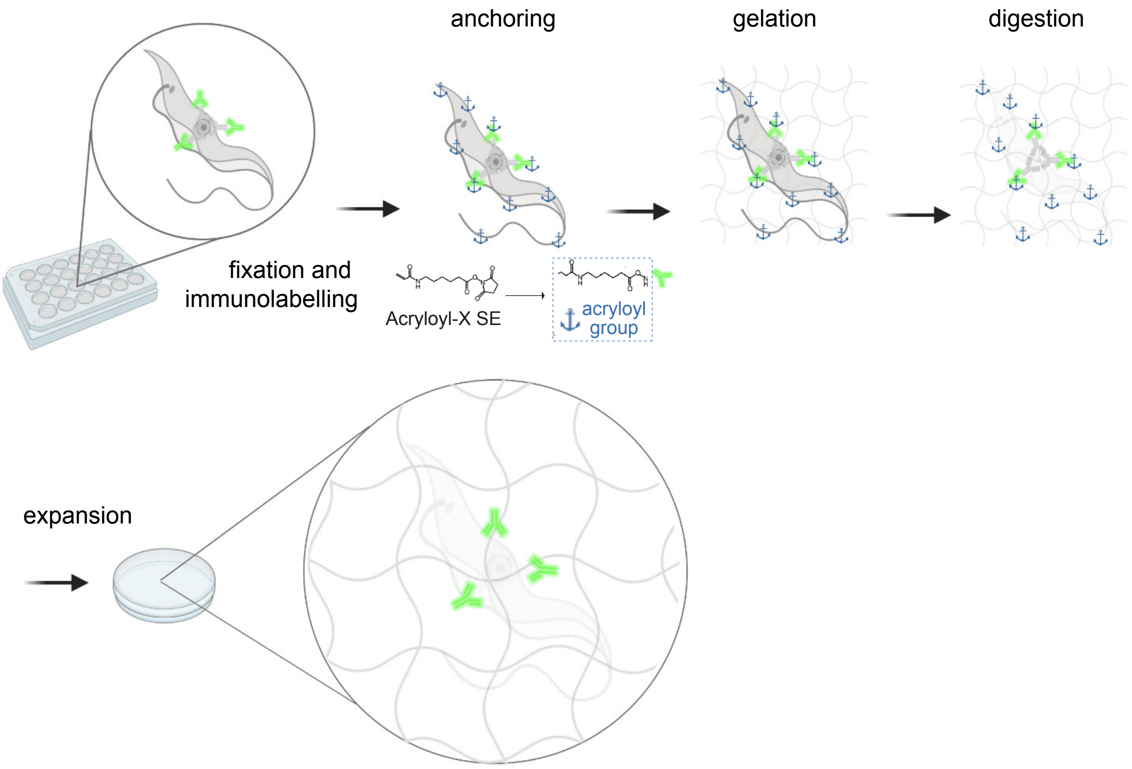

B principle of UExM

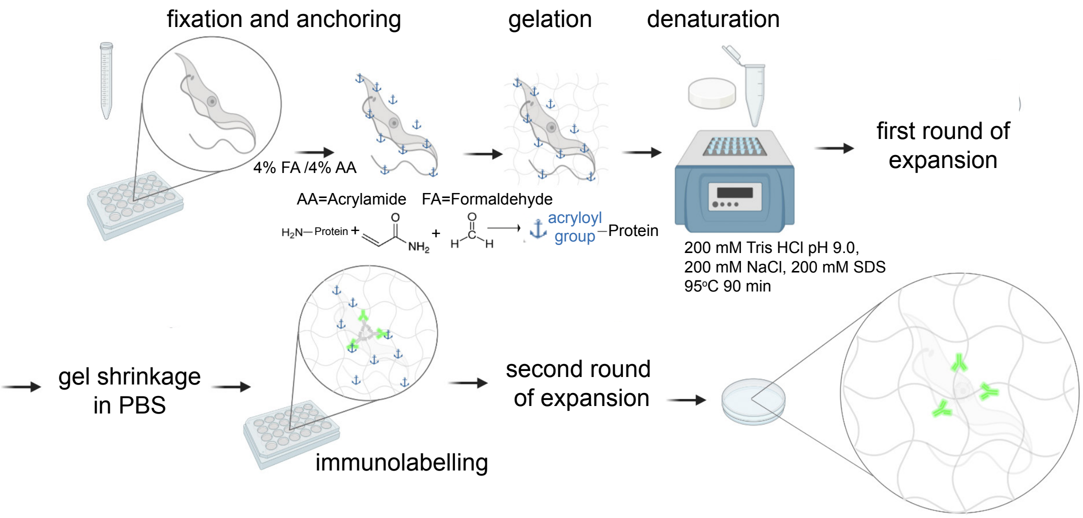

C Expansion factor

ProExM

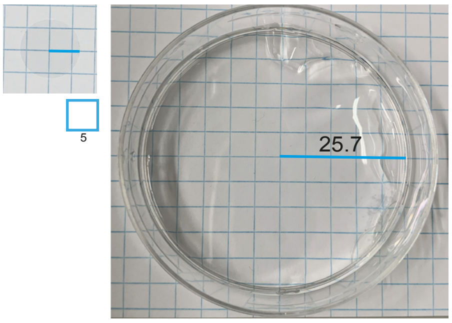

25.7 ÷ 7 = **3.6**

UExM

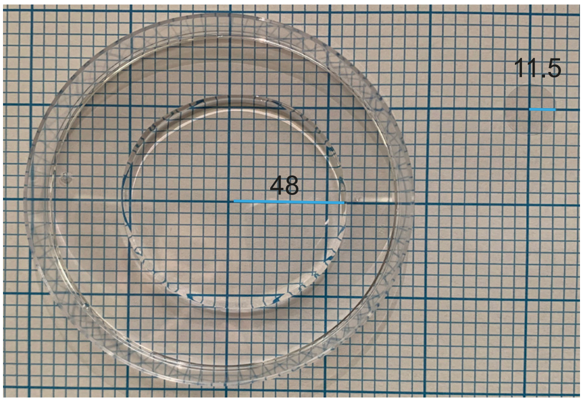

48 ÷ 11.5 = **4.2**

D Test for isotropic expansion

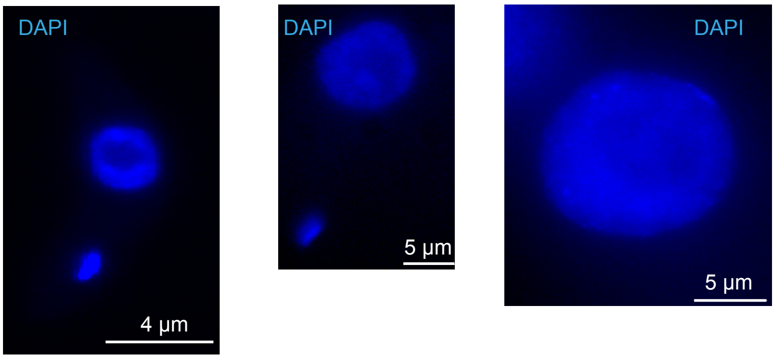

	no expansion	proExM	UExM
example image:	(image)	(image)	(image)
diameter of nuclei ± SD:	2.4 ± 0.2 µm (n = 21)	8.4 ± 0.7 µm (n =72)	11.5 ± 1.1 µm (n = 52)
expansion factor based on nuclei:		8.4 ÷ 2.5 = **3.5**	11.5 ÷ 2.5 = **4.6**

Figure S1: Validation of Protein Retention Expansion Microscopy (proExM) and Ultrastructural Expansion Microscopy (UExM) (A) Workflow of the proExM method, adapted from[1]. **(B)** Workflow of the UExM method, adapted from[2]. **(C)** Calculation of the expansion factor for proExM (left) and UExM (right) by comparing the size of the non-expanded and the expanded gel, using squared paper as a size reference. Measurements were done with Fiji[3]. **(D)** The diameter of DAPI stained nuclei was measured in unexpanded cells and after expansion. The so-measured expansion factor is very similar to the one measured by the increase in gel size (C), indicating isotropic expansion of the nucleus.
Some images were created in BioRender (https://biorender.com/b03t383).

[1]Asano SM, Gao R, Wassie AT, Tillberg PW, Chen F & Boyden ES (2018) Expansion Microscopy: Protocols for Imaging Proteins and RNA in Cells and Tissues. Curr Protoc Cell Biol 80
[2]Gambarotto D, Hamel V & Guichard P (2021) Ultrastructure expansion microscopy (U-ExM). In pp 57–81.
[3]Schindelin J, Arganda-Carreras I, Frise E, Kaynig V, Longair M, Pietzsch T, Preibisch S, Rueden C, Saalfeld S, Schmid B, et al (2012) Fiji: an open-source platform for biological-image analysis. Nat Methods 9: 676–682
