# Supplementary material for: Detailed characterisation of the trypanosome nuclear pore architecture reveals conserved asymmetrical functional hubs that drive mRNA export
Source: PLoS Biol. 2025 Feb 3;23(2):e3003024. doi: 10.1371/journal.pbio.3003024 (PMC11825100; doi:10.1371/journal.pbio.3003024)
Supplement: S2 Fig — (PDF) [file pbio.3003024.s002.pdf]

Figure S2

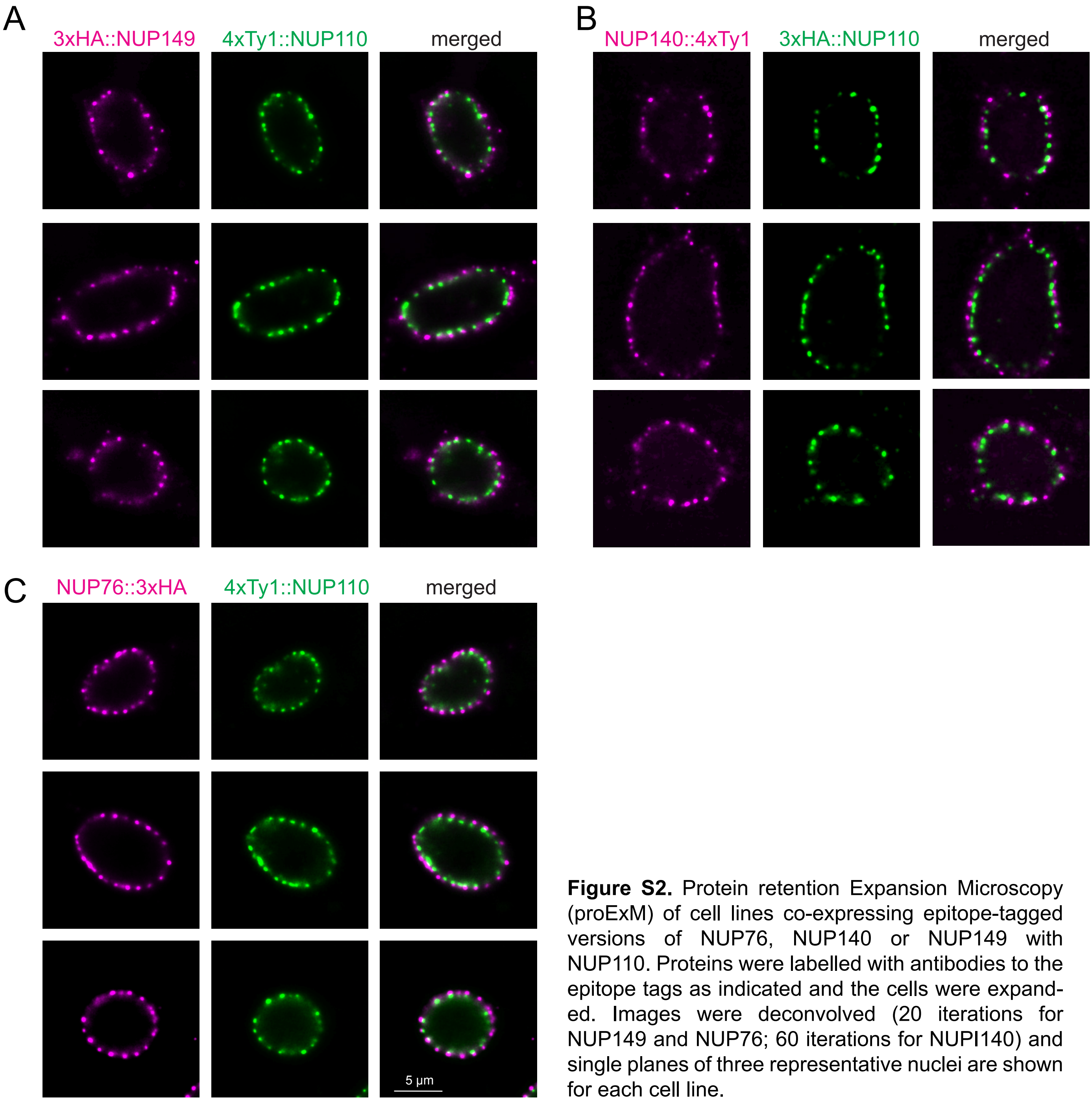

Figure S2. Protein retention Expansion Microscopy (proExM) of cell lines co-expressing epitope-tagged versions of NUP76, NUP140 or NUP149 with NUP110. Proteins were labelled with antibodies to the epitope tags as indicated and the cells were expanded. Images were deconvolved (20 iterations for NUP149 and NUP76; 60 iterations for NUPI140) and single planes of three representative nuclei are shown for each cell line.
