# Supplementary material for: Detailed characterisation of the trypanosome nuclear pore architecture reveals conserved asymmetrical functional hubs that drive mRNA export
Source: PLoS Biol. 2025 Feb 3;23(2):e3003024. doi: 10.1371/journal.pbio.3003024 (PMC11825100; doi:10.1371/journal.pbio.3003024)
Supplement: S3 Fig — (PDF) [file pbio.3003024.s003.pdf]

Figure S3

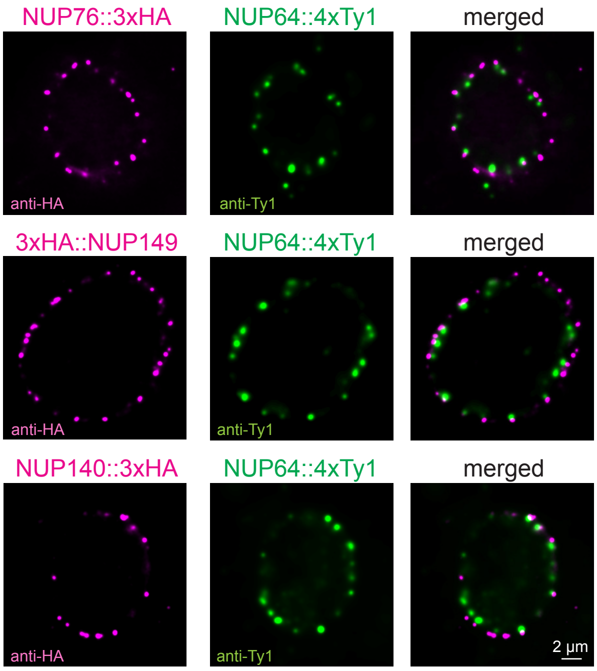

Figure S3. Ultrastructure Expansion Microscopy (UExM) of lines co-expressing 3xHA-tagged versions of NUP76/NUP140/NUP149 with NUP64::4xTy1. Labelling was done with anti-Ty1 and anti-HA. Images were deconvolved with 60 iterations and a single plane image of one nucleus of each cell line is shown.
