# Supplementary material for: Detailed characterisation of the trypanosome nuclear pore architecture reveals conserved asymmetrical functional hubs that drive mRNA export
Source: PLoS Biol. 2025 Feb 3;23(2):e3003024. doi: 10.1371/journal.pbio.3003024 (PMC11825100; doi:10.1371/journal.pbio.3003024)
Supplement: S4 Fig — (PDF) [file pbio.3003024.s004.pdf]

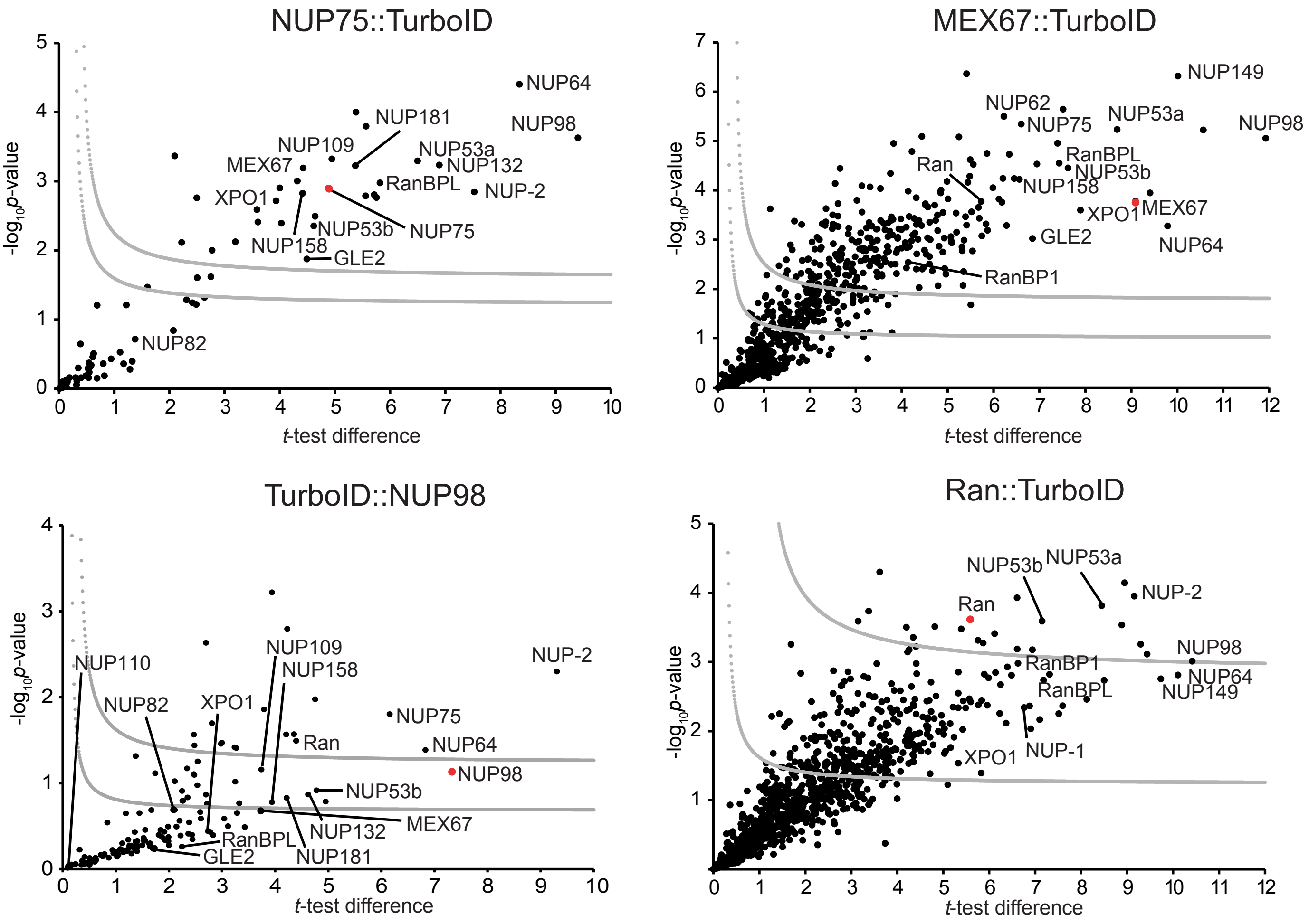

Figure S4: Statistical analysis of TurboID experiments.
Hawaii plot (multiple volcano plots) of label-free quantification results of the BioID experiments for NUP75, Ran and MEX67 with fused TurboID tag at the C-terminus and NUP98 with fused TurboID tag at the N-terminus . All samples were prepared at least in duplicate. To generate the volcano plots, the $-\log_{10}p$-value was plotted versus the *t*-test difference (difference between means), comparing each respective bait experiment to the wt control. Potential interactors were classified according to their position in the plot, applying cut-off curves for “significant class A” (SigA; gray, upper curve; FDR = 0.01, s0 = 0.1) and “significant class B” (SigB; gray, lower curve; FDR = 0.05, s0 = 0.1), respectively. Bait proteins are indicated by a red dot, selected known NUPs and transport factors are labelled and LFQ data is given in Table S2.
