# Supplementary material for: Detailed characterisation of the trypanosome nuclear pore architecture reveals conserved asymmetrical functional hubs that drive mRNA export
Source: PLoS Biol. 2025 Feb 3;23(2):e3003024. doi: 10.1371/journal.pbio.3003024 (PMC11825100; doi:10.1371/journal.pbio.3003024)
Supplement: S5 Fig — (PDF) [file pbio.3003024.s005.pdf]

Figure S5

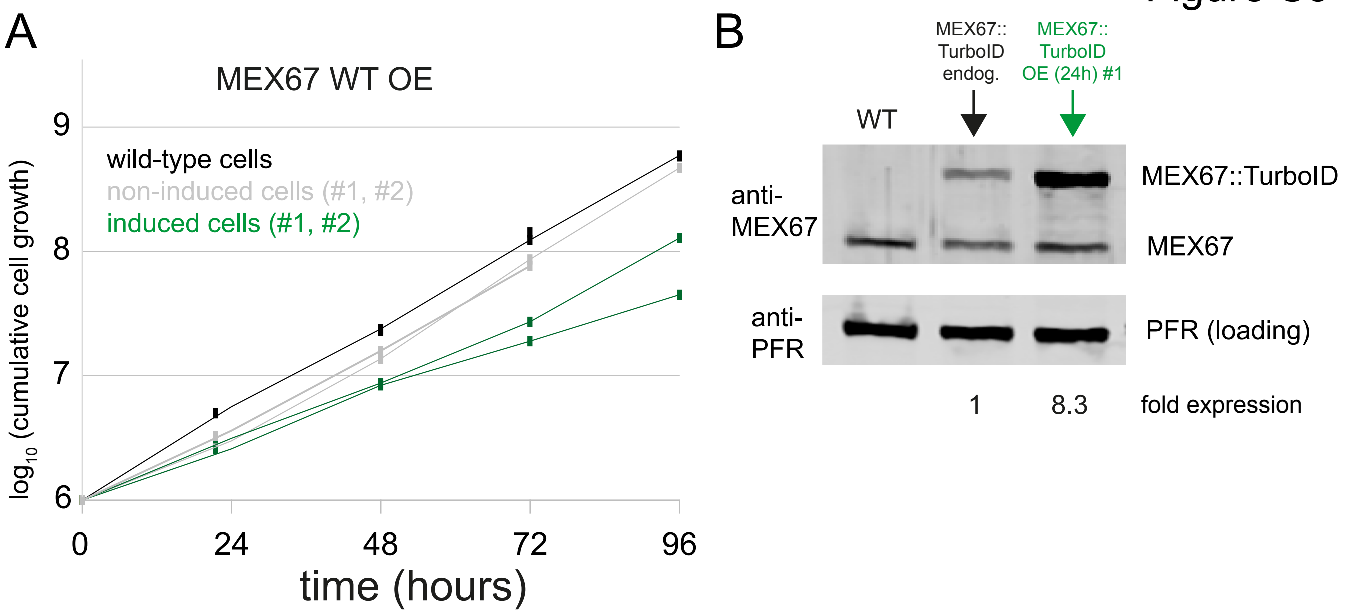

Figure S5. Inducible overexpression of MEX67::TurboID
(A) Growth of wild-type cells and of two clones with the inducible expression of Mex67::TurboID from an ectopic locus without (gray) and with (green) induction using tetracyclin. Growth was monitored for 96 hours with daily measurements. Raw data can be found in Table S3. (B) Western blot loaded with cell lysates of wild-type cells, cells expressing MEX67::TurboID from the endogenous locus and cells with induced expression of MEX67::TurboID from an ectopic locus for 24 hours (clone #1). The Western blot was probed with antibodies specific to MEX67 and to PFR (to control for loading). The intensities of the bands were quantified using hte LiCor Odyssey software and the extent of overexpression is shown, normalised by the loading control.
