# Supplementary figures and images for: Detailed characterisation of the trypanosome nuclear pore architecture reveals conserved asymmetrical functional hubs that drive mRNA export
Source: PLoS Biol. 2025 Feb 3;23(2):e3003024. doi: 10.1371/journal.pbio.3003024 (PMC11825100; doi:10.1371/journal.pbio.3003024)

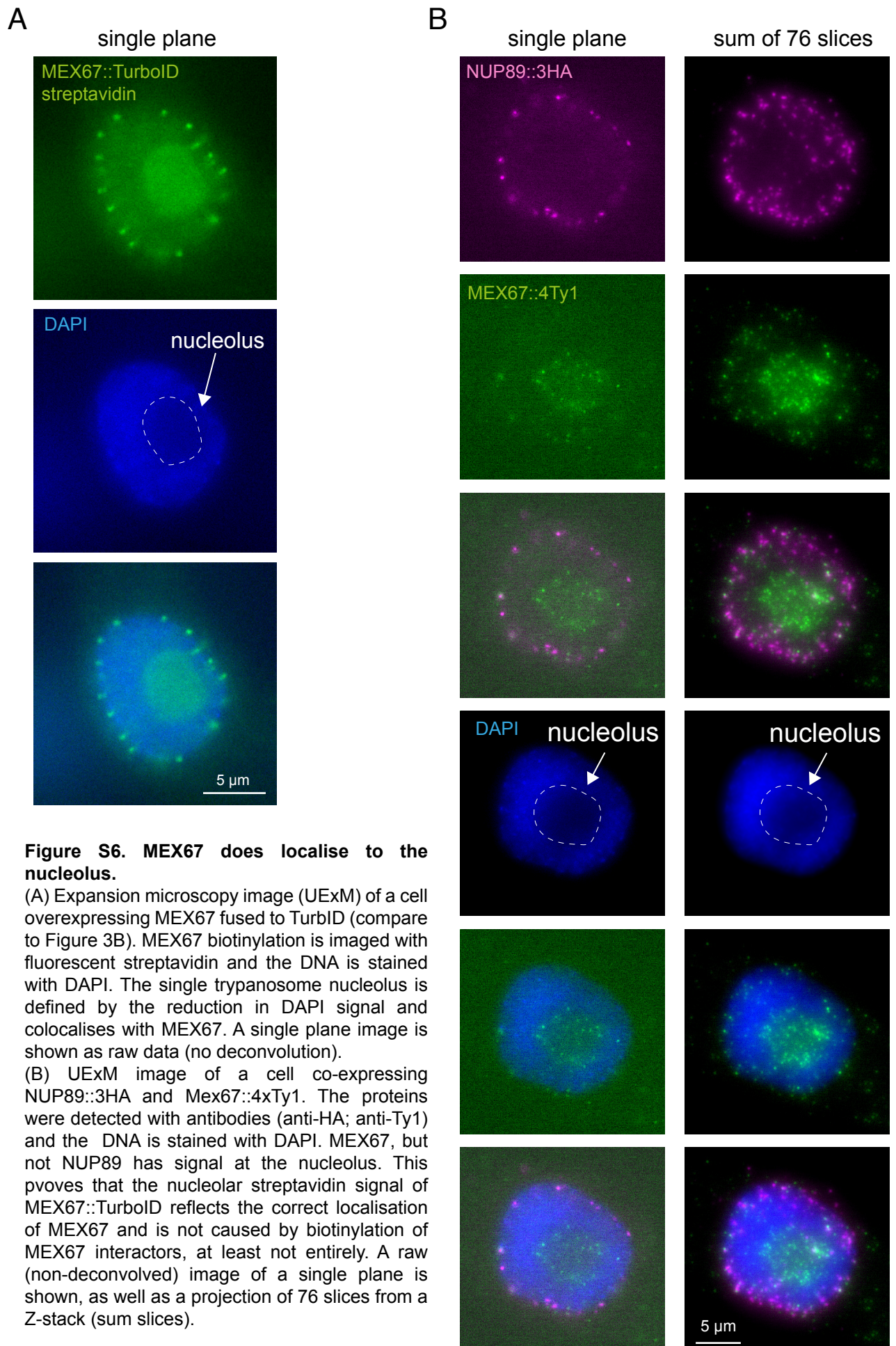

Supplement: S6 Fig — (PDF) [file pbio.3003024.s006.pdf]

Figure S15

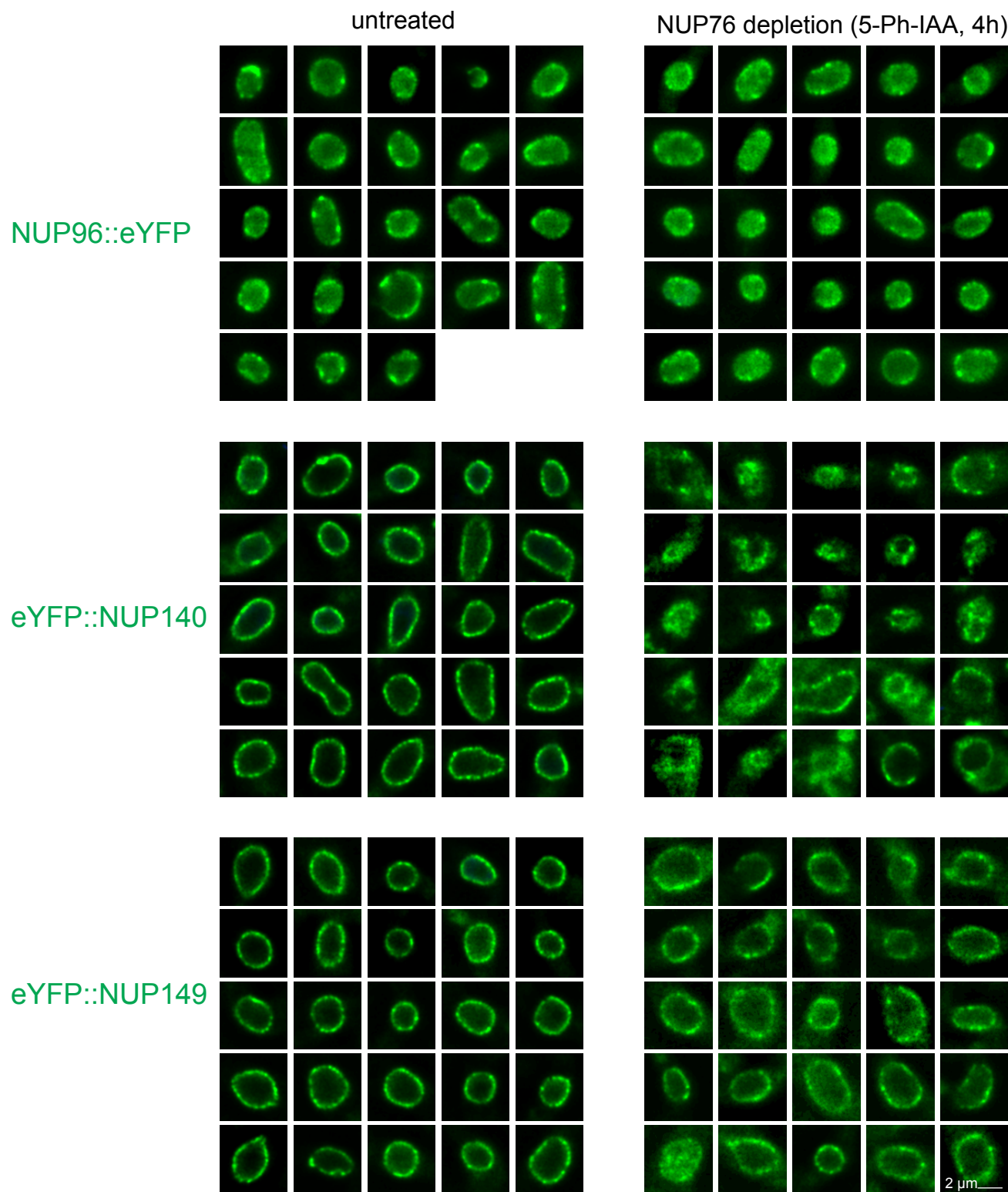

**Figure S15:** Additional images for Figure 6D and E.

Supplement: S15 Fig — (PDF) [file pbio.3003024.s015.pdf]
