# Supplementary material for: Detailed characterisation of the trypanosome nuclear pore architecture reveals conserved asymmetrical functional hubs that drive mRNA export
Source: PLoS Biol. 2025 Feb 3;23(2):e3003024. doi: 10.1371/journal.pbio.3003024 (PMC11825100; doi:10.1371/journal.pbio.3003024)
Supplement: S7 Fig — (PDF) [file pbio.3003024.s007.pdf]

Figure S7 page 1/2

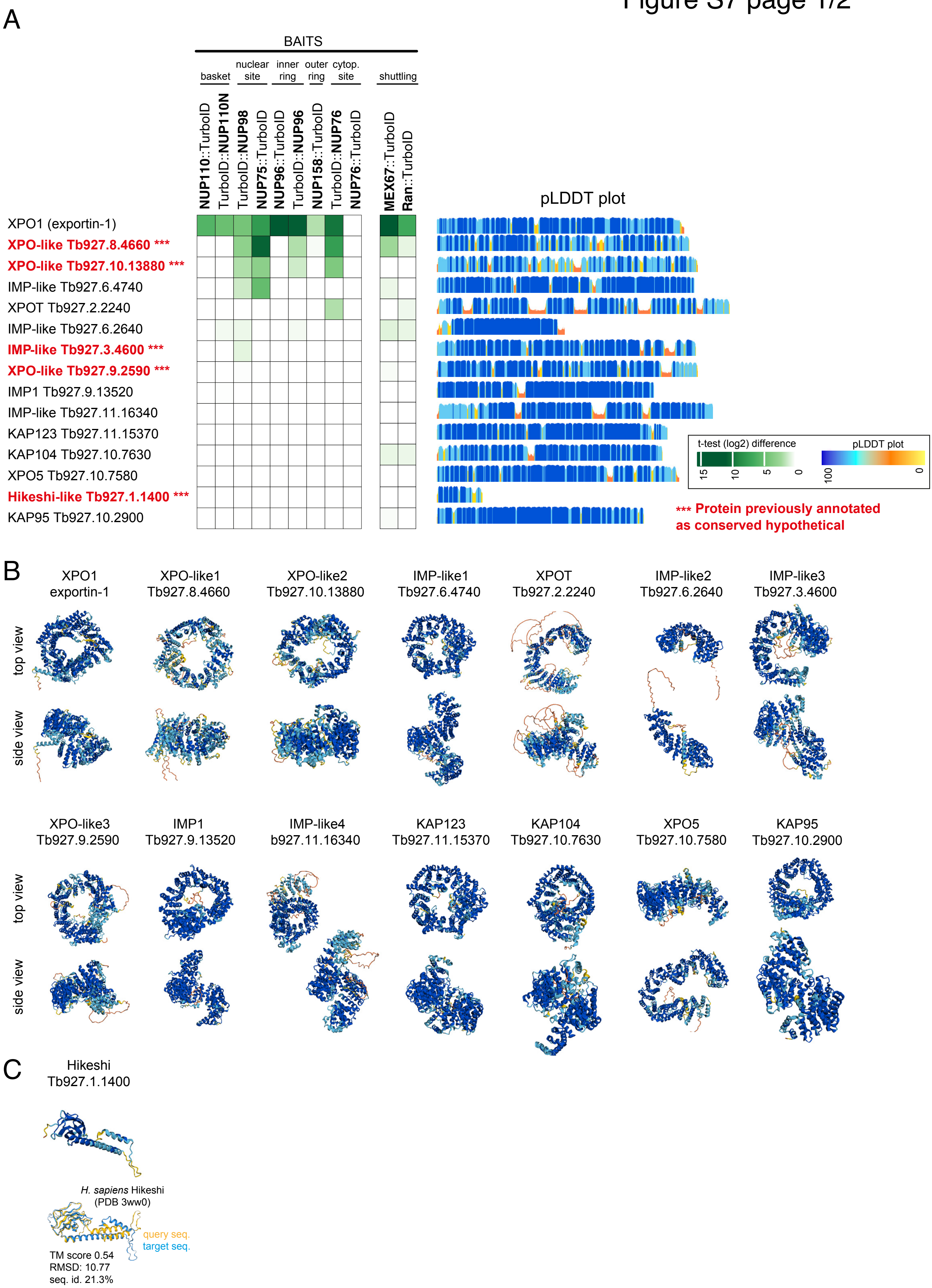

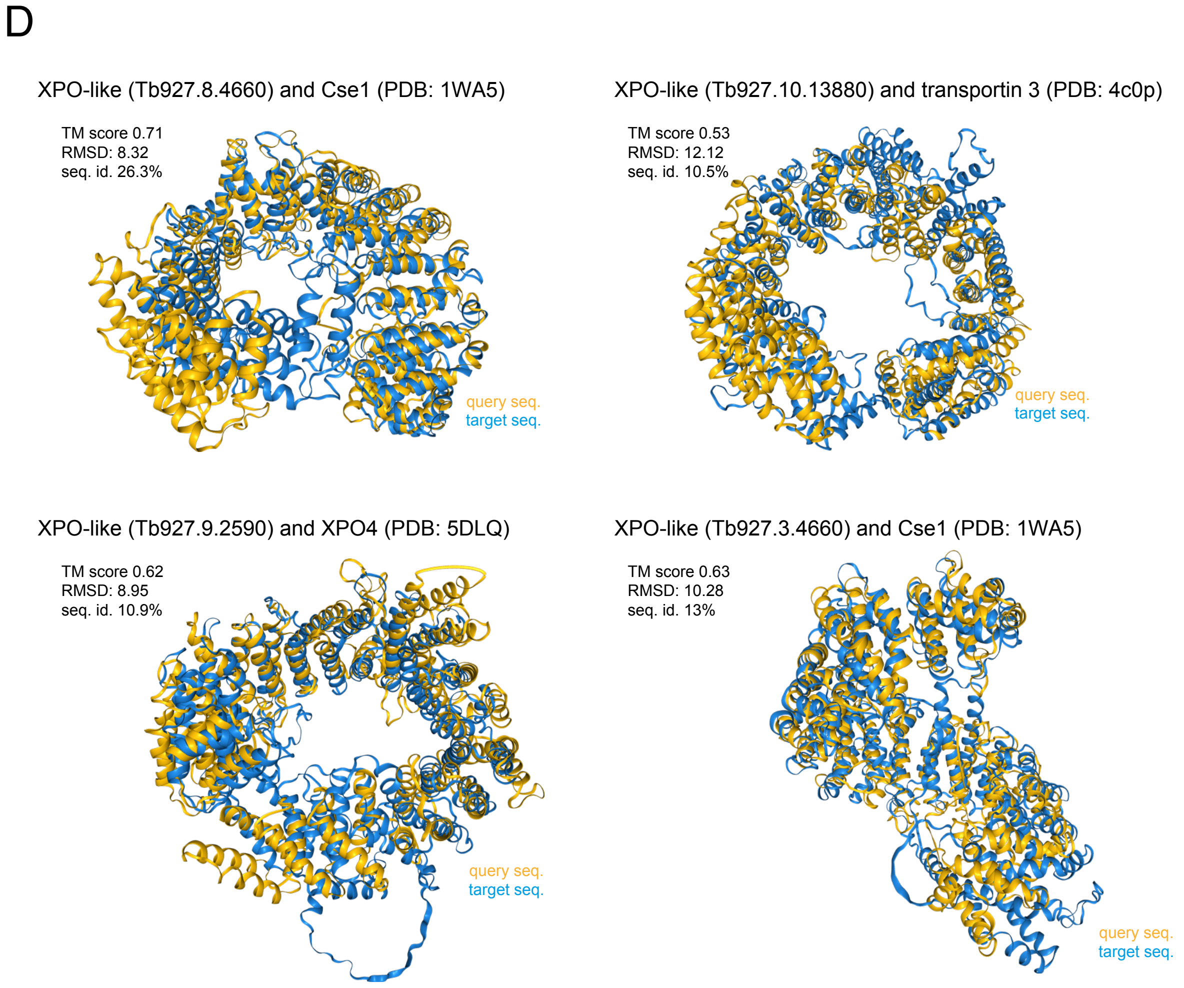

Figure S7: Karyopherins are poorly labelled by our proximity map
(A) All trypanosome proteins with nuclear pore localisation predicted by TrypTag[1] were screened for the presence of importin/exportin folds by running their AlphaFold2 models[2] in FoldSeek[3]. This resulted in the identification of 15 importins/exportins, of which five were not previously annoted as such (***** in A**). Mass spectrometry data from proximity labelling experiments of the indicated baits were analysed and the labelling of these karyopherins is shown with a color-code (t-test difference values, log2-transformed). pLLDT plots are included. Details on the mass spectrometry data can be found in Table S1.

(B) Trypanosmatid-optimised AlphaFold2 models of 14 karyopherins (top and side view of the models, taken from[2] and coloured based on pLLDT values shown in (A).

(C) Trypanosmatid-optimised AlphaFold2 models of Hikeshi (taken from[2] and coloured based on pLLDT values shown in (A) and the FoldSeek outputs[3]: superimposition between the AlphaFold2 models of the trypanosomatid orthologues (target sequence, coloured blue) and the FoldSeek best hit on the PDB database (query sequence, coloured yellow), with root mean square deviation of atomic positions (RMSD) of the superimposition, internal confidence values (template modelling scores, TM, ranging from 0-1, from worst to best) and sequence identity values below.

(D) The four proteins with structural homologies to karyopherins that were not annotated as karyopherins (***** in A**) and their FoldSeek search outputs[3]: superimposition between the AlphaFold2 models of the trypanosomatid orthologues (target sequence, coloured blue) and the FoldSeek best hit on the PDB database (query sequence, coloured yellow), with root mean square deviation of atomic positions (RMSD) of the superimposition, internal confidence values (template modelling scores, TM, ranging from 0-1, from worst to best) and sequence identity values below.

[1] Billington, K. et al. Genome-wide subcellular protein map for the flagellate parasite Trypanosoma brucei. Nat. Microbiol. 8, 533–547 (2023).
[2] Wheeler R. A resource for improved predictions of Trypanosoma and Leishmania protein three-dimensional structure. PLoS ONE 16(11): e0259871 (2021).
[3] van Kempen, M. et al. Fast and accurate protein structure search with Foldseek. Nat Biotechnol. 42, 243–246 (2024).
