# Supplementary material for: Detailed characterisation of the trypanosome nuclear pore architecture reveals conserved asymmetrical functional hubs that drive mRNA export
Source: PLoS Biol. 2025 Feb 3;23(2):e3003024. doi: 10.1371/journal.pbio.3003024 (PMC11825100; doi:10.1371/journal.pbio.3003024)
Supplement: S8 Fig — (PDF) [file pbio.3003024.s008.pdf]

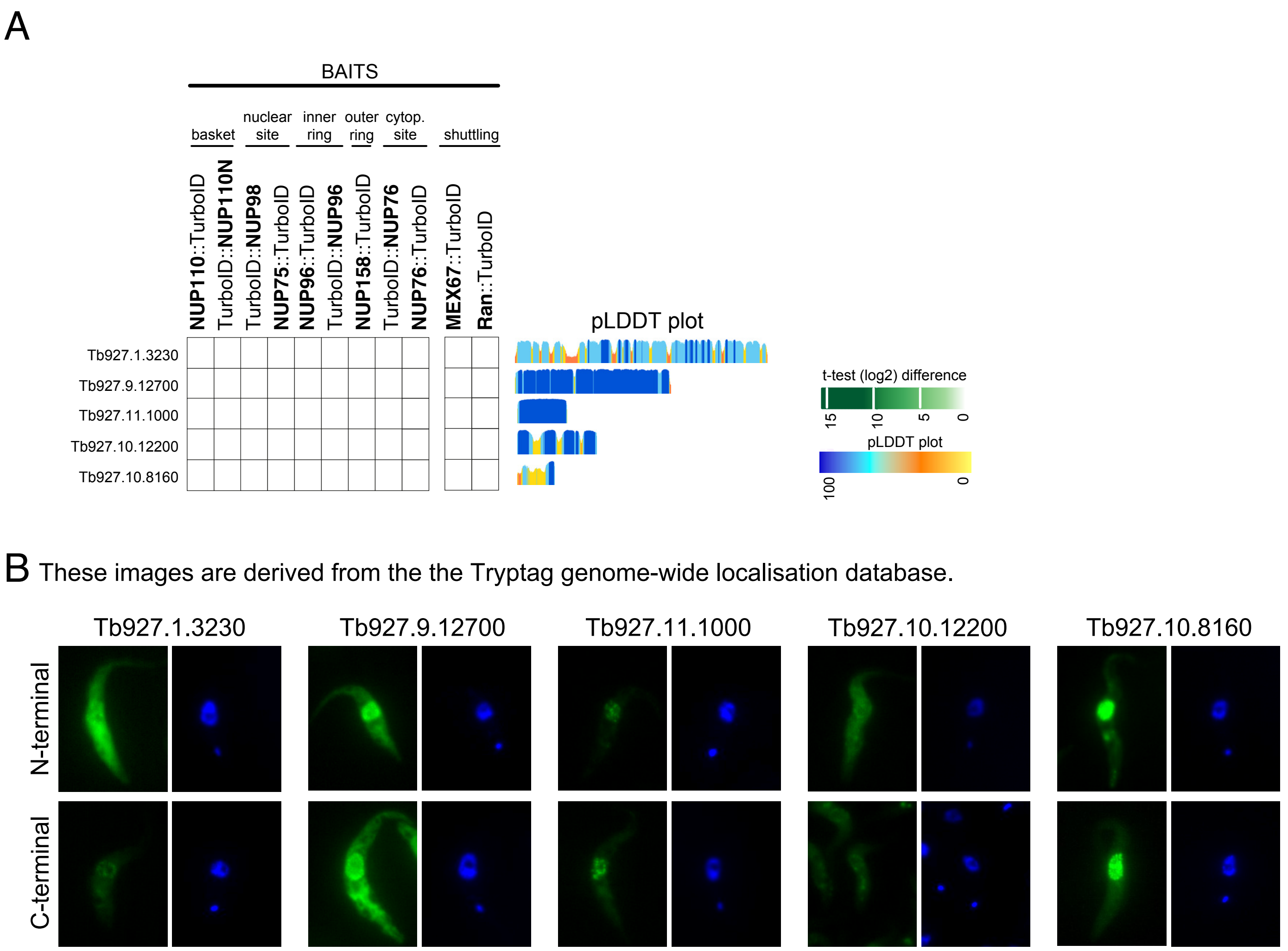

Figure S8: Proteins with nuclear pore localisation that are not labelled by either of the baits of our proximity map
(A) Of all proteins with predicted nuclear pore localisation by TrypTag[a], five were not labelled by either of the baits from our proximity map, and the pLDDT plots are shown.
(B) Localisation images of mNeongreen fusions of these proteins were taken from TrypTag[a]. The DNA is stained with DAPI and shown in blue. Images of both N-terminal and C-terminal mNeongreen fusions are shown.

[a] Billington, K. et al. Genome-wide subcellular protein map for the flagellate parasite Trypanosoma brucei. Nat. Microbiol. 8, 533–547 (2023).
