# Supplementary material for: Detailed characterisation of the trypanosome nuclear pore architecture reveals conserved asymmetrical functional hubs that drive mRNA export
Source: PLoS Biol. 2025 Feb 3;23(2):e3003024. doi: 10.1371/journal.pbio.3003024 (PMC11825100; doi:10.1371/journal.pbio.3003024)
Supplement: S9 Fig — (PDF) [file pbio.3003024.s009.pdf]

Figure S9

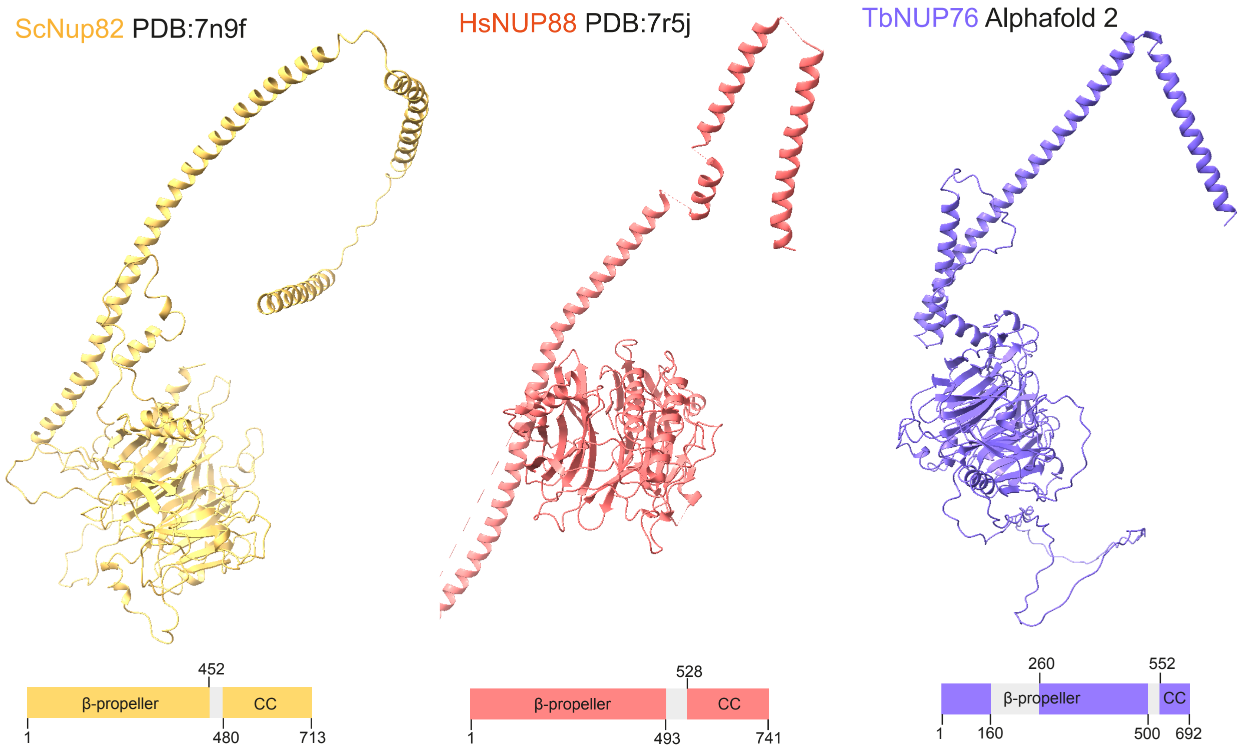

Figure S9: Homologies between experimentally resolved structures of ScNup82[1], HsNUP88[2] and the AlphaFold2-predicted model of TbNUP76[3]. All three proteins have very similar structures, namely an N-terminal beta-propeller that is followed by coiled-coil (CC) domains. One difference is the long disordered region within the beta-propeller of TbNUP76, that is not present in *Sc*NUP82 and HsNUP88. Below each structure is a schematic drawing.

1 Akey CW, Singh D, Ouch C, Echeverria I, Nudelman I, Varberg JM, Yu Z, Fang F, Shi Y, Wang J, et al (2022) Comprehensive structure and functional adaptations of the yeast nuclear pore complex. Cell 185: 361-378.e25

2 Mosalaganti S, Obarska-Kosinska A, Siggel M, Taniguchi R, Turoňová B, Zimmerli CE, Buczak K, Schmidt FH, Margiotta E, Mackmull M-T, et al (2022) AI-based structure prediction empowers integrative structural analysis of human nuclear pores. Science (1979) 376

3 Wheeler RJ (2021) A resource for improved predictions of Trypanosoma and Leishmania protein three-dimensional structure. PLoS One 16: e0259871
