# Supplementary material for: Detailed characterisation of the trypanosome nuclear pore architecture reveals conserved asymmetrical functional hubs that drive mRNA export
Source: PLoS Biol. 2025 Feb 3;23(2):e3003024. doi: 10.1371/journal.pbio.3003024 (PMC11825100; doi:10.1371/journal.pbio.3003024)
Supplement: S10 Fig — (PDF) [file pbio.3003024.s010.pdf]

Figure S10

A

NUP149 zinc fingers

```
ZnF1   24  KDAWVCTRCGKVKDLRGDHLRGKMEVRSDCWPCGKKATFKLQSR  69
ZnF2  136  SSVWKCSICGKVKPLSGDHLRGKTEVRSDCWPCGCKRTFVLSPV 179
ZnF3  466  KDAWVCTRCGKVKDLRGDHLRGKMEVRSDCWPCGKKATFKLQSR 509
ZnF4  576  SSVWKCSTCGKVKPLSGDHLRGKTEVRSDCWPCGCKRTFVLSPV 619
ZnF5  786  KDAWVCTRCGKVKDLRGDHLRGKMEVRSDCWPCGKKATFKLQSR 829
ZnF6  896  SSVWKCSTCGKVKPLSGDHLRGKTEVRSDCWPCGCKRTFVLSPV 939
           ...* *: ***** * ******* ********** * ** *.

TbNUP149 ZnF       W-C-RCGKVK-L-GDHLRGK-EVRSDCWPCG-KATF-L
human NUP153 ZnF  W-C--C---N------C--C
human NUP358 ZnF  W-C--C---N------C--C
```

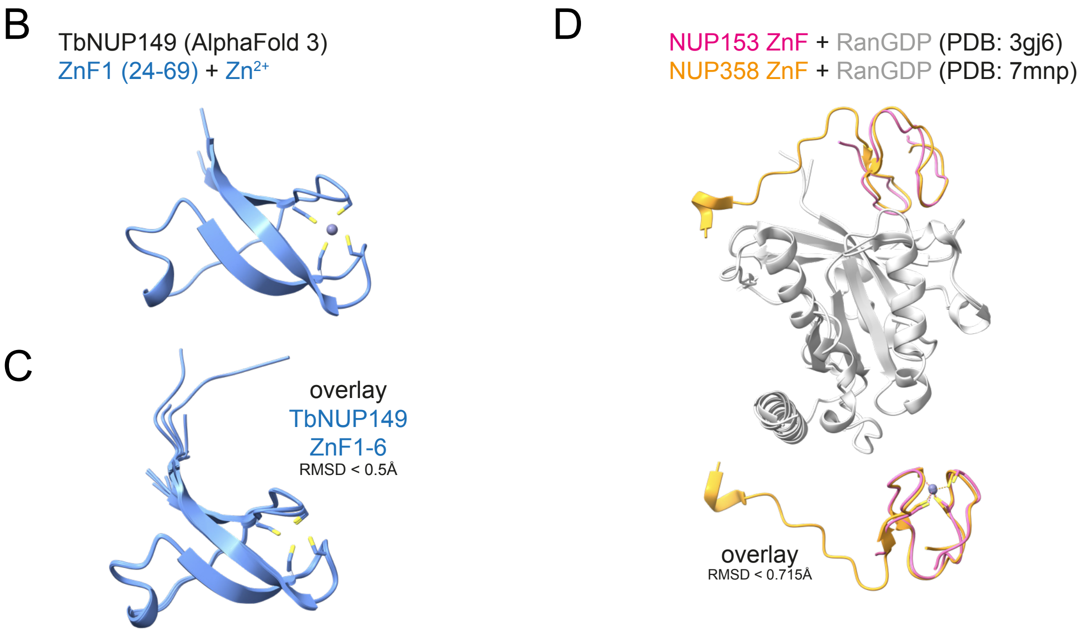

Figure S10:

(A) Alignment of the six zinc fingers of *T. brucei* NUP149 with consensus sequence. The consensus sequence of the unrelated zinc fingers of the human NUP153 and NUP358 (taken from [1]) is shown for comparison. Arrows point to the cysteine residues used to chelate the Zn^{2+} ion (shown in B).

(B) AlphaFold3[2] model of the most N-terminal zinc finger of NUP149 (Zn1, amino acids 24-69) together with a zinc ion. The cysteine side chains are shown in yellow.

(C) Trypanosome-optimised AlphaFold2 models of all six zinc fingers of NUP149 were superimposed, using the AlphaFold2 structure of the first zinc finger as a reference structure (RMSD of all superimpositions are all below 0.5Å). All cysteine side chains are shown in yellow.

(D) Overlay of the experimentally resolved structures of the zinc fingers of human NUP153[3] and human NUP358[4] in complex with RanGDP. Note that there is no similarity to the zinc fingers of *T. brucei* NUP149 (compare B and C).

[1] Partridge, J. R. & Schwartz, T. U. Crystallographic and Biochemical Analysis of the Ran-binding Zinc Finger Domain. J. Mol. Biol. 391, 375–389 (2009).
[2] Abramson, J. et al. Accurate structure prediction of biomolecular interactions with AlphaFold 3. Nature 630, 493–500 (2024).
[3] Partridge, J. R. & Schwartz, T. U. Crystallographic and Biochemical Analysis of the Ran-binding Zinc Finger Domain. J. Mol. Biol. 391, 375–389 (2009).
[4] Bley, C. J. et al. Architecture of the cytoplasmic face of the nuclear pore. Science 376, eabm9129 (2022).
