# Supplementary material for: Detailed characterisation of the trypanosome nuclear pore architecture reveals conserved asymmetrical functional hubs that drive mRNA export
Source: PLoS Biol. 2025 Feb 3;23(2):e3003024. doi: 10.1371/journal.pbio.3003024 (PMC11825100; doi:10.1371/journal.pbio.3003024)
Supplement: S11 Fig — (PDF) [file pbio.3003024.s011.pdf]

Figure S11

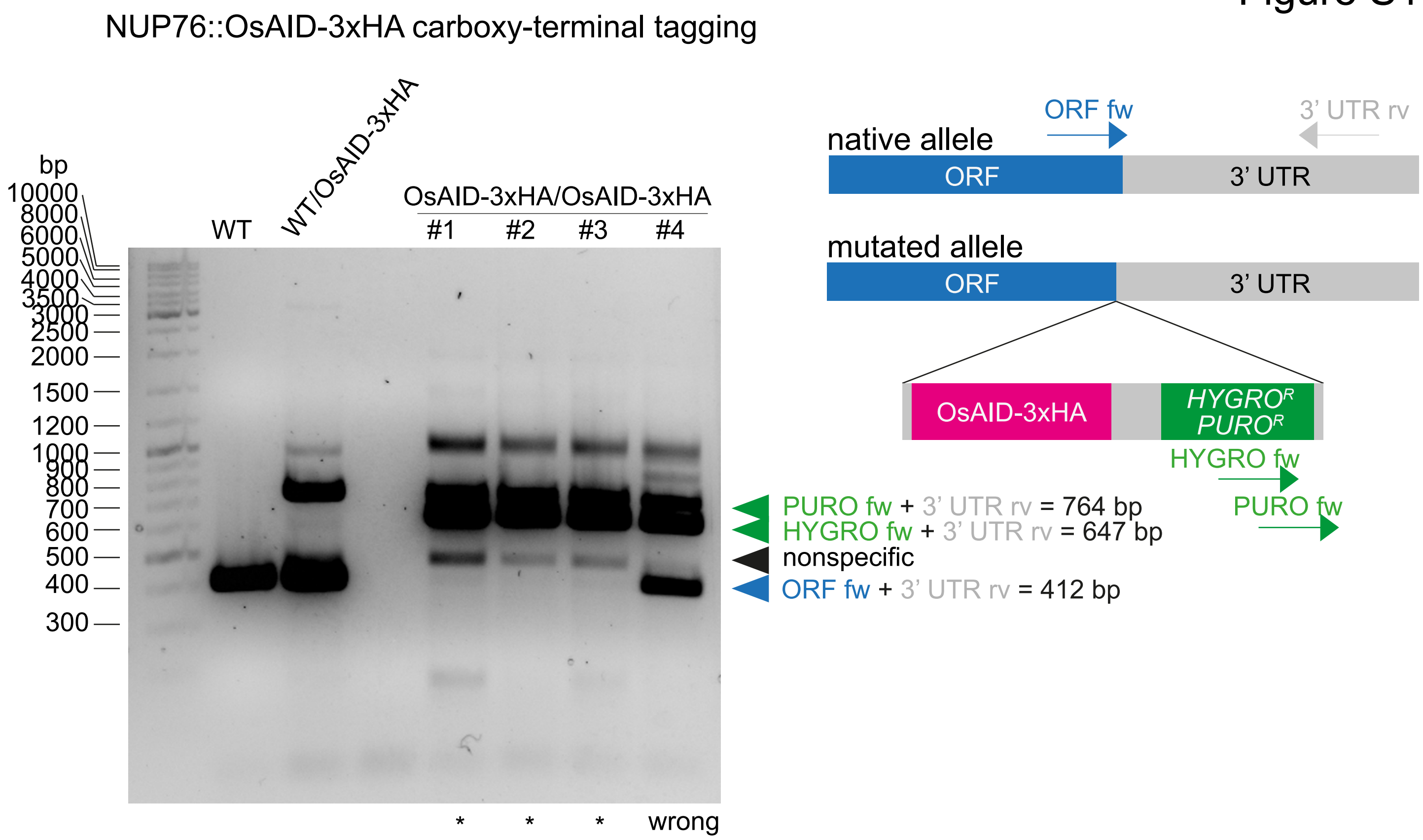

Figure S11: Confirmation of NUP76::OsAID-3xHA homozygous cell lines by diagnostic PCR

(A) The auxin inducible degron system was employed for inducible degradation of *T. brucei* NUP76. Both endogenous alleles of the NUP76 gene were fused to OsAID-3xHA at the C-terminus. Two resistance cassettes were used, one with a puromycin and the other with a hygromycin resistance gene. The PCR strategy that was used to evaluate the cell line and, in particular, to control for the absence of the wild type allele, is schematically pictured (right). PCR reactions were performed with a mixture of all three forwards oligos and the reverse oligo and products are resolved on an agarose gel. A non-specific band is indicated with a black triangle. The wild type and the mutated bands are indicated with a blue and green triangles, respectively. Three clones are positive (#1-3, labelled with *) and (#4) is wrong.
