# Supplementary material for: Detailed characterisation of the trypanosome nuclear pore architecture reveals conserved asymmetrical functional hubs that drive mRNA export
Source: PLoS Biol. 2025 Feb 3;23(2):e3003024. doi: 10.1371/journal.pbio.3003024 (PMC11825100; doi:10.1371/journal.pbio.3003024)
Supplement: S12 Fig — Additional poly(A) FISH images and fluorescence profiles of NUP76 depleted cells. (PDF) [file pbio.3003024.s012.pdf]

Figure S12

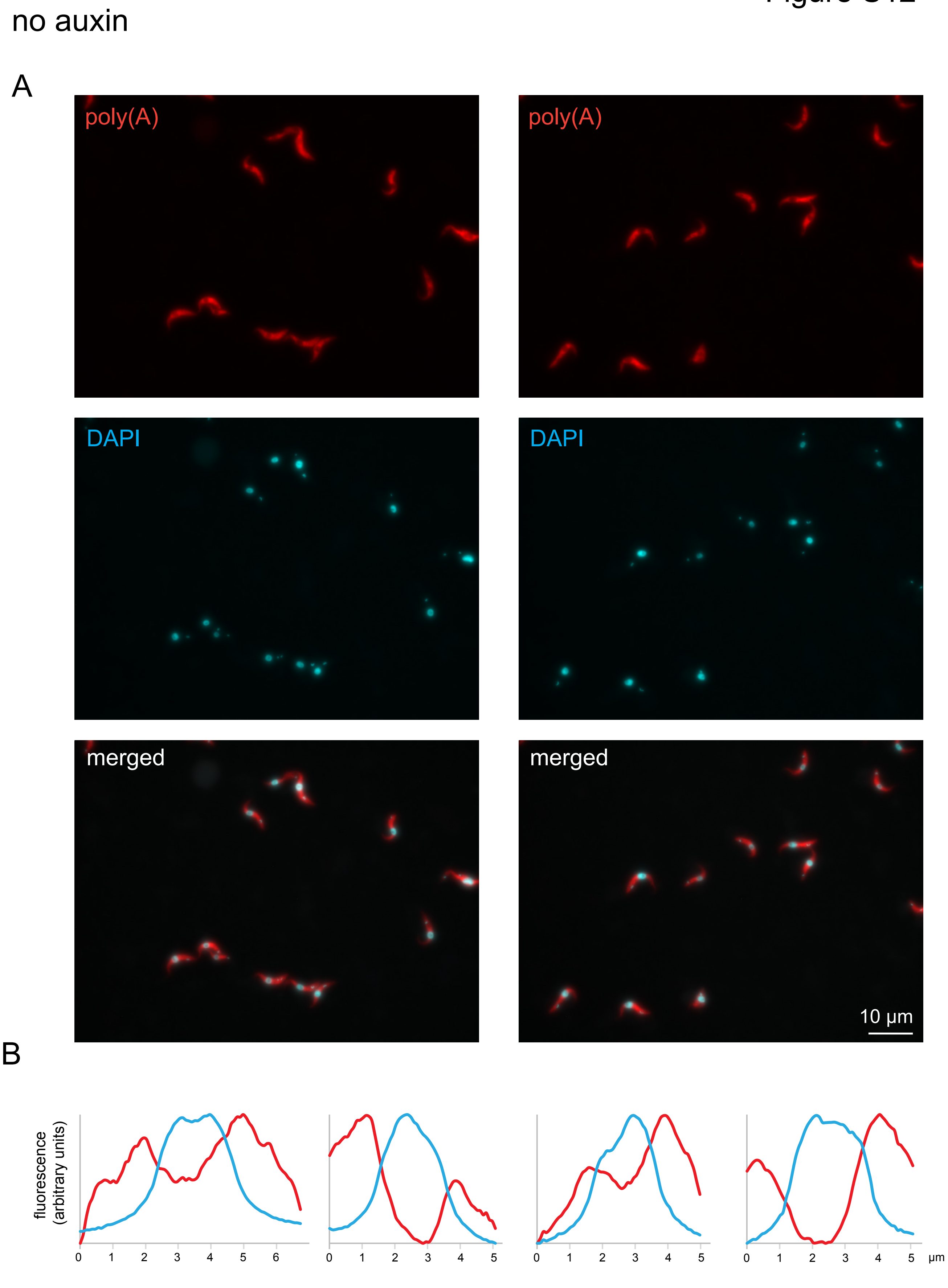

Figure S12: ***In situ*** **hybridisation of untreated cells.**
Cells were probed with oligo dT to monitor mRNA localisation. The DNA is labelled with DAPI. **(A)** Images are presented as sum-slices of 75 images recorded at 140 nm distance.
(B) For 4 nuclei, we show fluorescence profiles through the nucleus, to demonstrate cytoplasmic poly(A) signal. Raw data are in Table S4.
