# Supplementary material for: Detailed characterisation of the trypanosome nuclear pore architecture reveals conserved asymmetrical functional hubs that drive mRNA export
Source: PLoS Biol. 2025 Feb 3;23(2):e3003024. doi: 10.1371/journal.pbio.3003024 (PMC11825100; doi:10.1371/journal.pbio.3003024)
Supplement: S13 Fig — Additional poly(A) FISH images and fluorescence profiles of NUP76 depleted cells. (PDF) [file pbio.3003024.s013.pdf]

NUP76 depletion: 2 h auxin

Figure S13

A

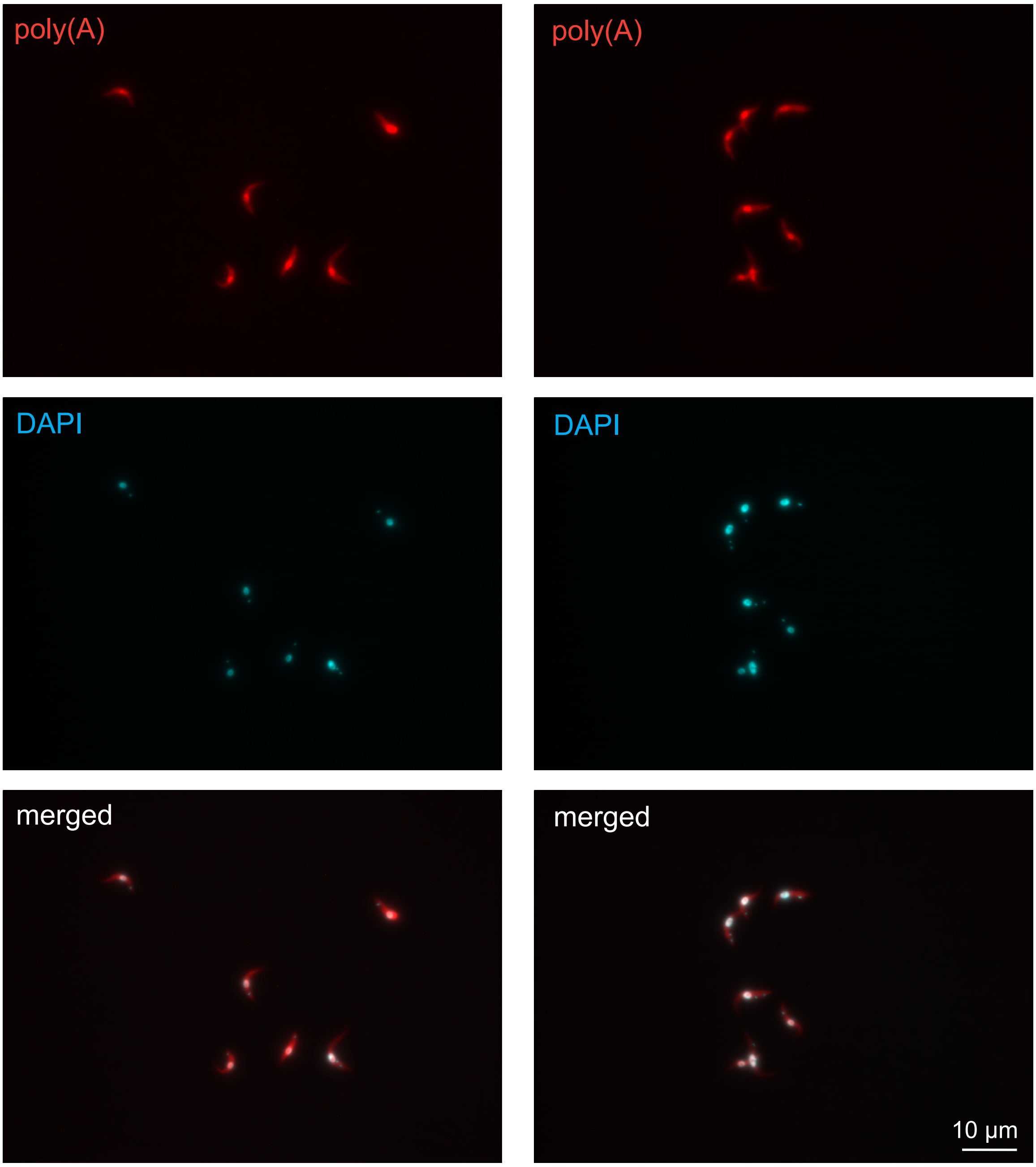

B

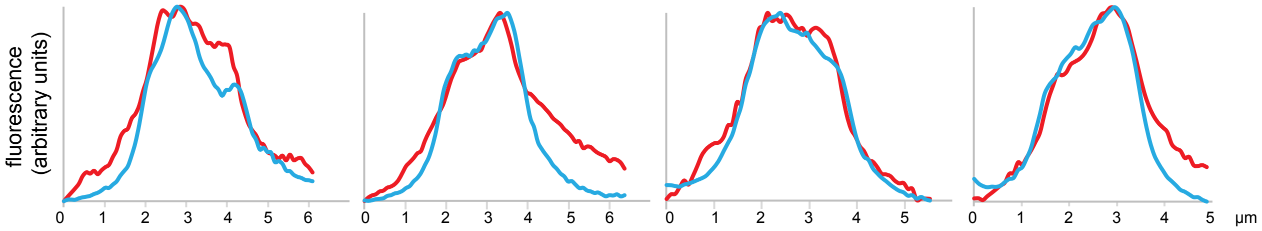

DAPI
poly(A)

Figure S13: *In situ* hybridisation of cells after NUP76 depletion (2 hours auxin). Cells were probed with oligo dT to monitor mRNA localisation. The DNA is labelled with DAPI. (A) Images are presented as sum-slices of 75 images recorded at 140 nm distance.
(B) For 4 nuclei, we show fluorescence profiles through the nucleus, to demonstrate nuclear poly(A) signal. Raw data are in Table S4.
