# Supplementary material for: Detailed characterisation of the trypanosome nuclear pore architecture reveals conserved asymmetrical functional hubs that drive mRNA export
Source: PLoS Biol. 2025 Feb 3;23(2):e3003024. doi: 10.1371/journal.pbio.3003024 (PMC11825100; doi:10.1371/journal.pbio.3003024)
Supplement: S14 Fig — Additional poly(A) FISH images and fluorescence profiles of NUP76 depleted cells. (PDF) [file pbio.3003024.s014.pdf]

Figure S14

NUP76 depletion: 4 h auxin

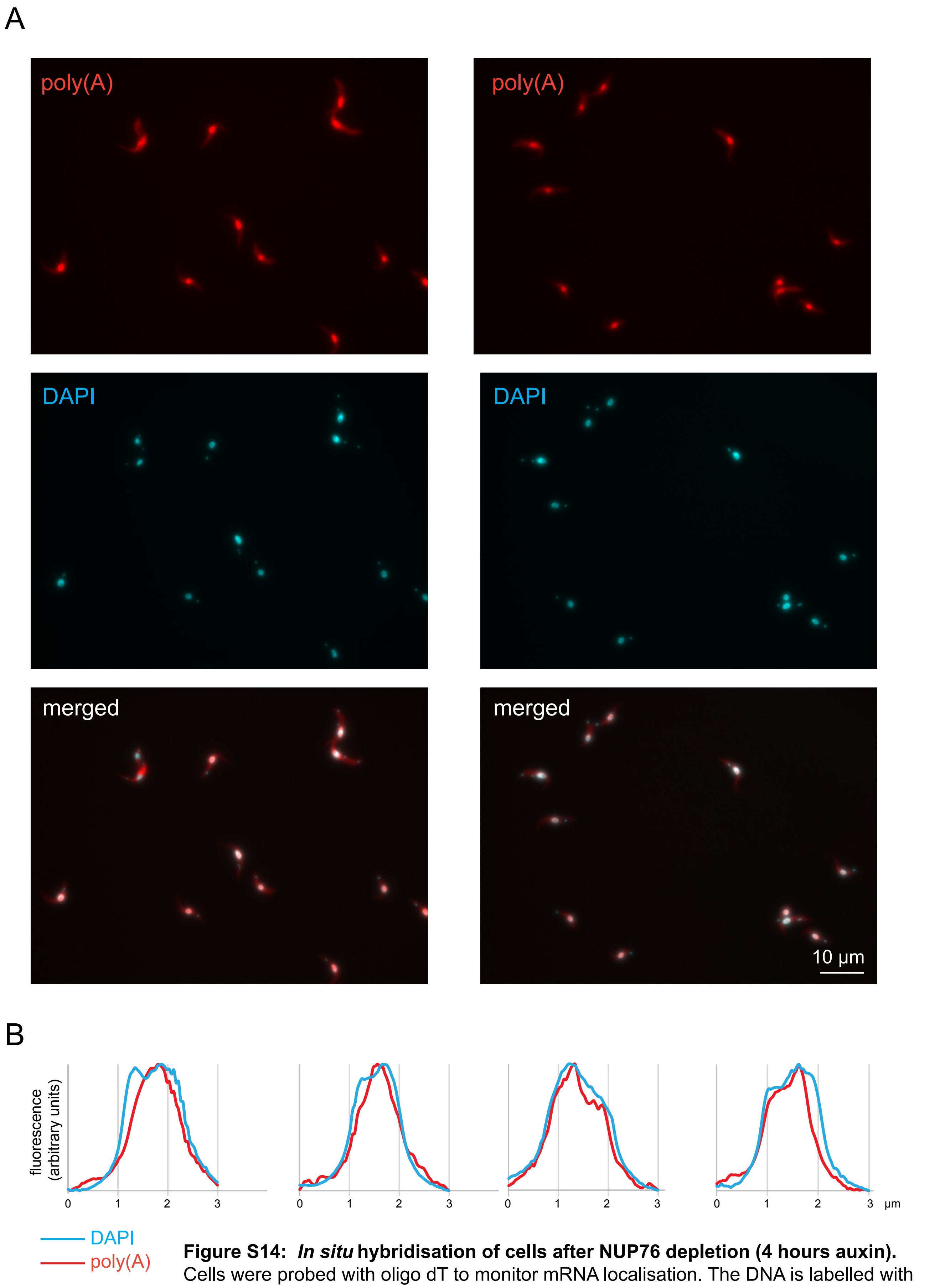


**Figure S14: *In situ* hybridisation of cells after NUP76 depletion (4 hours auxin).** Cells were probed with oligo dT to monitor mRNA localisation. The DNA is labelled with DAPI. **(A)** Images are presented as sum-slices of 75 images recorded at 140 nm distance. **(B)** For 4 nuclei, we show fluorescence profiles through the nucleus, to demonstrate nuclear poly(A) signal. Raw data are in Table S4.
