# Supplementary material for: Detailed characterisation of the trypanosome nuclear pore architecture reveals conserved asymmetrical functional hubs that drive mRNA export
Source: PLoS Biol. 2025 Feb 3;23(2):e3003024. doi: 10.1371/journal.pbio.3003024 (PMC11825100; doi:10.1371/journal.pbio.3003024)
Supplement: S16 Fig — (PDF) [file pbio.3003024.s016.pdf]

Figure S16

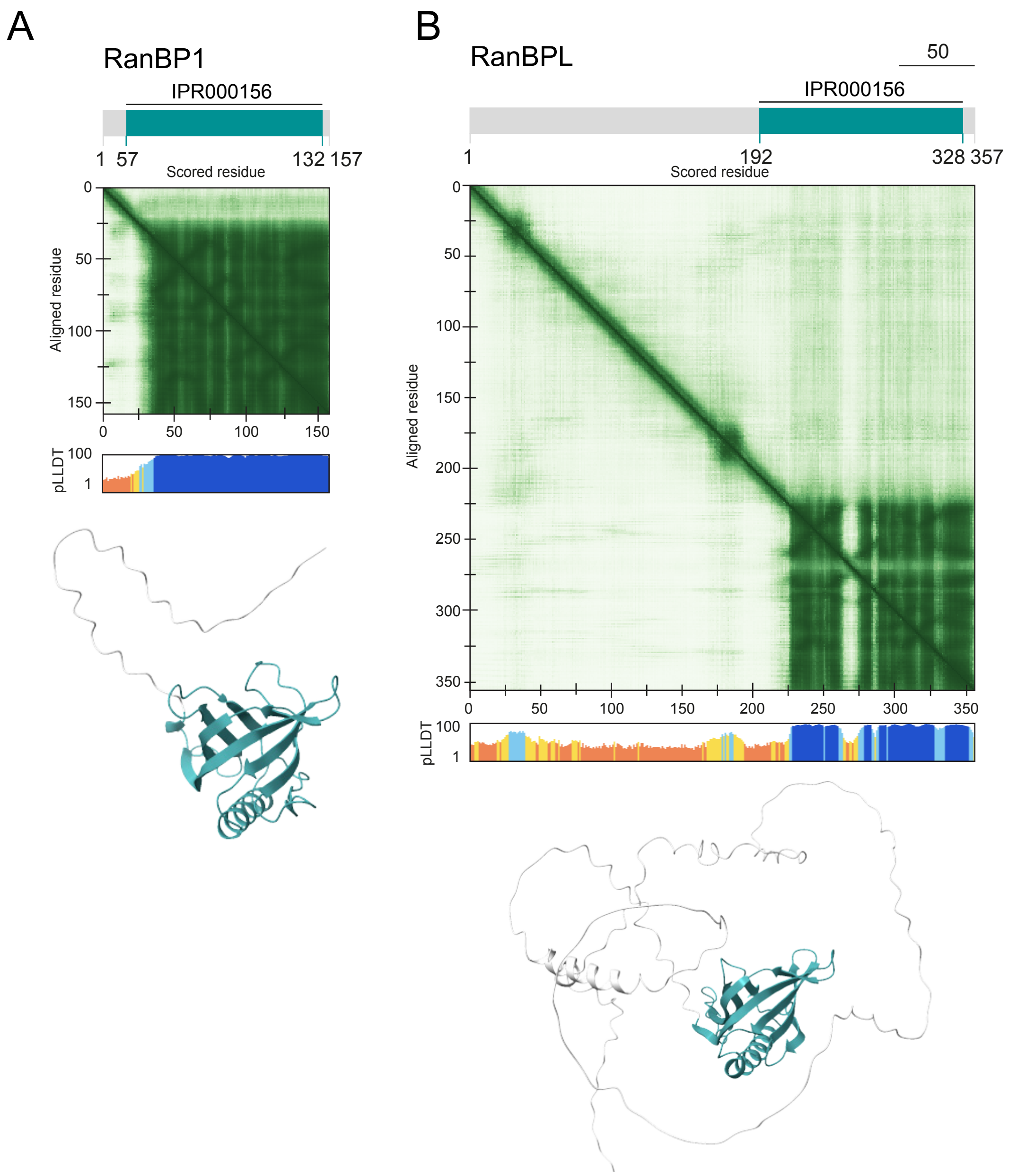

Figure S16: Trypanosome RanBP1 and RanBPL
Models of trypanosome-optimised AlphaFold2 predictions of RanBP1 **(A)** and RanBPL **(B)** pAE plots, PLLDT plots and the predicted structures are shown, with structured parts coloured and disordered regions shown in grey.
