# Supplementary material for: Detailed characterisation of the trypanosome nuclear pore architecture reveals conserved asymmetrical functional hubs that drive mRNA export
Source: PLoS Biol. 2025 Feb 3;23(2):e3003024. doi: 10.1371/journal.pbio.3003024 (PMC11825100; doi:10.1371/journal.pbio.3003024)

S1 raw images

Figure S5 raw data (colours)

Mex67 signal and quantification (Mex67-specific antibodies)

PFR signal and quantification (PFR-specific antibodies)

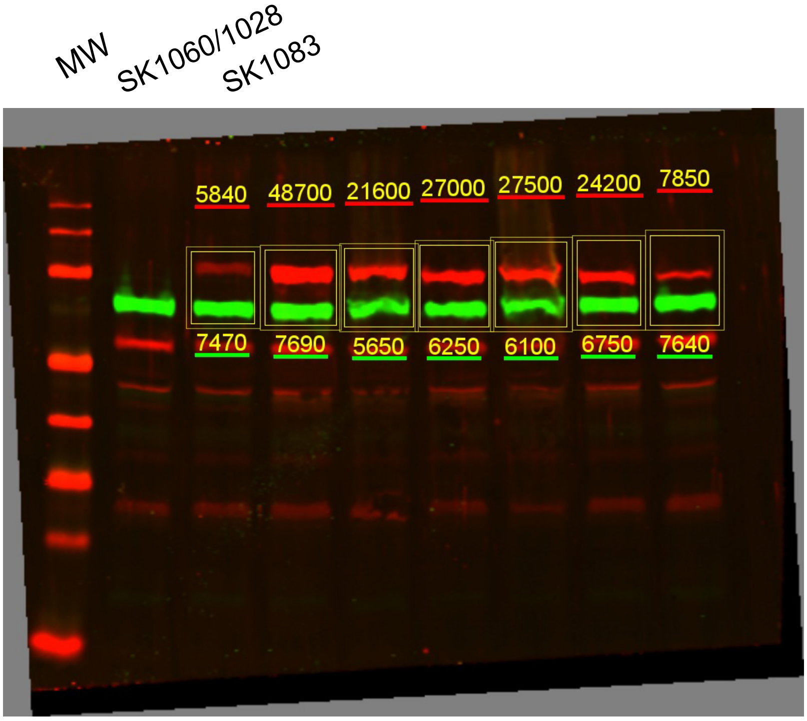

Quantification of Mex67/PFR signal

SK1060/1028 stands for Mex67::TurboID-Ty1 endogenous

SK1083 stands for Mex67::TurboID-Ty1 overexpression

	Mex67 signal	PFR signal	fold overexp.
SK1060/SK1028	5840	7470	
SK1083 #1 + tet 24h	48700	7690	8.3390411

Images were separated by colour channels and shown as gray scale

Figure S11 raw data

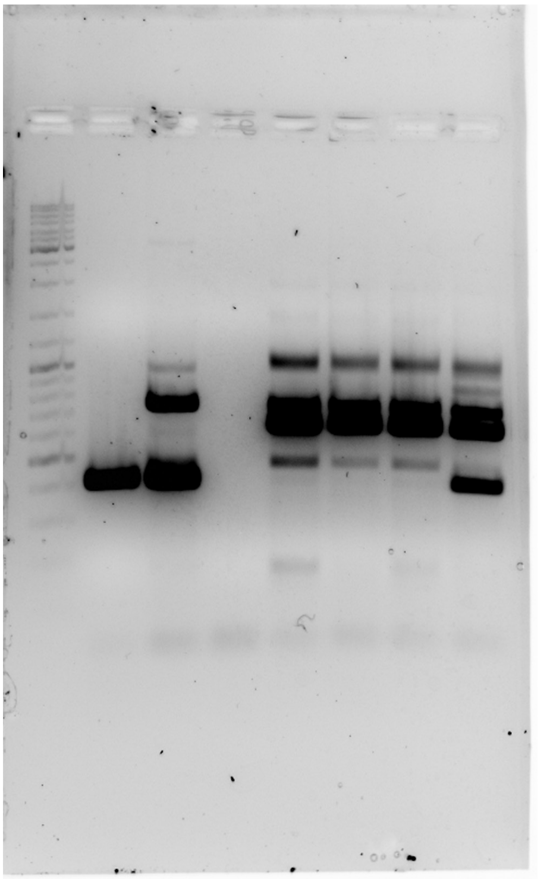

Figure S5 raw data (gray scale)

Mex67 (Mex67-specific antibodies)

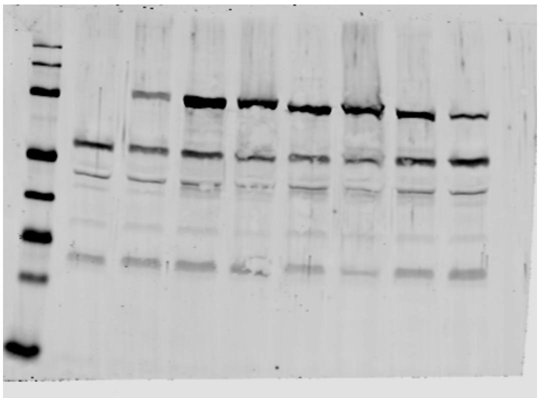

PFR (PFR-specific antibodies)

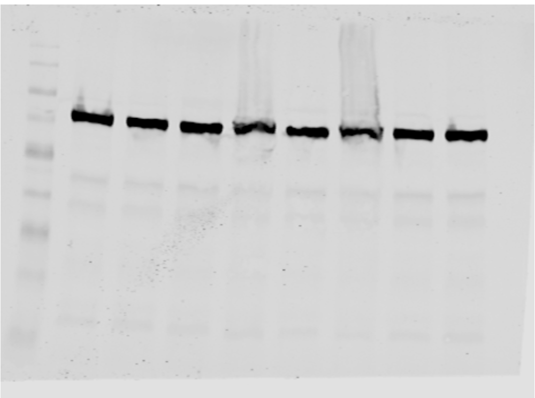

Figure 6A raw data

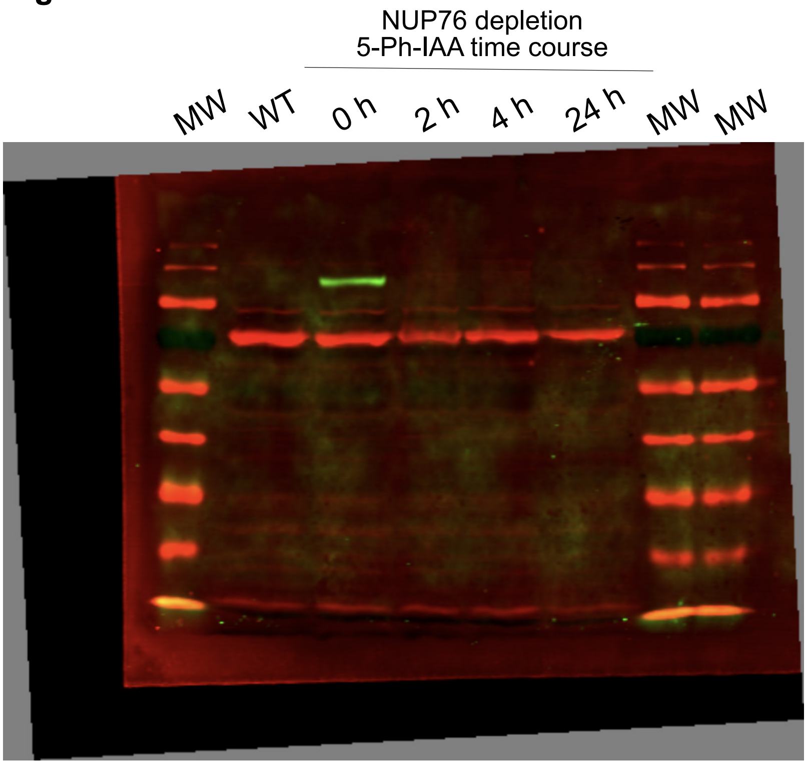

Supplement: S1 Raw Images — (PDF) [file pbio.3003024.s017.pdf]
