# Supplementary material for: Detailed characterisation of the trypanosome nuclear pore architecture reveals conserved asymmetrical functional hubs that drive mRNA export
Source: PLoS Biol. 2025 Feb 3;23(2):e3003024. doi: 10.1371/journal.pbio.3003024 (PMC11825100; doi:10.1371/journal.pbio.3003024)
Supplement: Attachment — Submitted filename: Rebuttal Letter.pdf [file pbio.3003024.s022.pdf]

Dear Mrs Vazquez Hernandez,

We have now carefully looked at the comments from the reviewers, please find our answers below. We have rewritten the manuscript according to the suggestions (for example included a discussion part now). We have done triplicates for the FISH experiment. We have also included one more set of mass spectrometry data, to improve the resolution of our map. We think that the manuscript has significantly improved now, and we like to thank you and the reviewers for the valuable suggestions.

Many greetings

Susanne Kramer

PS: We didn't had a change in authors, its just that Johanna Odenwald is now Johanna Krenzer due to marriage.

Reviewer #1:

This study represents a notable step forward in understanding the molecular architecture of the nuclear pore in Trypanosoma brucei parasites, valuable for the basic understanding of nuclear pore evolution as trypanosomes are among the earliest branching lineages of eukaryotes.
The key experimental data are expansion microscopy analysis of nuclear pore protein colocalisation for a subset of nuclear pore proteins, a proximity labelling (BirA/BioID-based) analysis of proximity of proteins within the nuclear pore and an elegant application of the auxin degron system to show that NUP76 is critical for mRNA export.

The experimental work appears overall of good quality. The expansion microscopy, BioID and auxin degron data appear to be of high quality with good controls, and I can see that the major results are supported and the major conclusions broadly justified.

Some of the microscopy results are presented qualitatively, where a basic image analysis would allow rigorous statistical support. Notably the localisation of mRNA relative to the nucleus in Figure 6C, showing nuclear accumulation of mRNA on NUP76 depletion. As this is a key result, a quantitative analysis of nuclear vs. cytoplasmic signal in a good number of cells and preferably from multiple biological replicates would be better. Especially, as 3 clones were generated, but data from only one is shown.

We have now done the FISH experiments for all three clones and observed no difference. We now present images with more cells in supplementary Figures and these also include fluorescence profiles, that clearly show the shift of the Poly(A) signal from

the cytoplasm to the nucleus upon NUP76 depletion. We have added the below to the Figure legend of Figure 6:

"Fluorescent profiles and images with more cells are shown in supplementary Figure S12-S14. Representative data of one out of three clones is shown."

Figure 6D,E would similarly benefit from a quantitative analysis, although less obvious what that would be, and well mitigated by showing multiple example nuclei and the extended presentation in the paired supplemental figure.

As the reviewer points out, there is no obvious way to quantify these data and we had actually tried a few quantifications options and the results were not convincing. Therefore, we prefer to stick to our "mitigation", which is assessing a large number of nuclei as shown in the respective main and supplemental figures.

Quantitative microscopy analysis is applied inconsistently, ie. cell lines listed in Figure 1A have measured signal separation presented in 1B, while those in 1C-E and 3B-C do not. Only HA-TY colocalisation appears to have been analysed. Is there a reason why HA-streptavidin should not also be measured? I think it would add value.

Your comment prompted us to reconsider our conclusion from the quantification in Figure 1B (see also in response to reviewer 2, point 2). We decided to remove the illustration of the quantification (1B) and only mention the values in the text, with the only conclusion that the distance between the NUP76 complex and basket is >120 nm, which we believe is valid. We are concerned that the illustration suggests an accuracy that is probably not achieved by the method. While a student t-test indeed confirms that NUP149 is further away from NUP110 than NUP76 and NUP140, the differences caused by tag positions (N or C terminal) and/or minor differences in expansion between cells are likely larger than the differences that we measure here. For the conclusions of this paper, the relative position of the NUP76 complex proteins are not important, and we therefore prefer to entirely remove the quantification to avoid any confusion and misinterpretation.

However, the text has some significant issues.

There is no conventional discussion section, and the conclusions section does not do a balanced discussion/summary of the result. Prevalence of discussion material in the results generally becomes more problematic as one progresses through the results section. There are numerous paragraphs which are entirely discussion, with no references to figures or data. As a particularly bad example, there are almost two pages

of text in the results section with no reference to figures (lines 521 to 570). I got to this section and genuinely wondered if I had missed a "discussion" title.

However, the introduction, with comprehensive and clear referencing of previous work, is clear. The verbose and clear methods section is good, similarly figure legends are broadly clear.

The paper would greatly benefit from a clear conventional discussion section and moving of the long discussion-like paragraphs from the results to this new discussion section. Much of the extended discussion within the results seems to be about presence/absence of orthologs, structural features, and names of sequence/structure/functionally orthologous proteins in other species. Given a key impact of this work is understanding the evolution of the nuclear pore, summarising this information in a carefully structured table or matrix would be more informative than the very verbose text.

We have now added a proper discussion part, also in response to reviewer 3.

I wasn't able to find some key discussion points which seem to arise from this work, notably:
What properties of the previous immuno EM work might have spuriously given a symmetrical nuclear pore result, and an honest exploration of what limitations of the expansion microscopy/TurboID could exist which may have spuriously given an asymmetric nuclear pore result. My opinion is that the expansion microscopy data is likely to be more robust, however a full balanced discussion would bolster the claim of asymmetry in the trypanosome nuclear pore structure.
This is now the third or fourth complementary approach to generating a comprehensive "nuclear poreome" (following the intial systematic affinity purification, the genome wide tagging/TrypTag and the T. brucei LOPIT analysis). I realise there are comparisons made in the text, and one supplemental figure for comparison to tagging data, but a summary table would be very beneficial. Similarly, discussion about the potential methodological origins of inconsistencies.

We have now added a section to the beginning of the new discussion chapter, comparing the different methodologies with the respective pros and cons. We also discuss the potential reason why the immuno EM gave different results. We are not sure about the value of a summary table. We do provide updated models and our heatmaps, but for tables we would need to set arbitrary thresholds to define localisations, which we would rather avoid.

“This study is an update of the trypanosome nuclear pore composition and is based on the foundations set by earlier studies: In 2009 (DeGrasse *et al*, 2009) had identified all trypanosomes NUPs using predicted structural similarities in pre-AlphaFold times. In 2016, Obado and colleagues have used a combination of high stringency precipitation of cryomilled cell material and electron microscopy with immune gold labelling to present the first detailed model of the trypanosome pore (Obado *et al*, 2016). Finaly, the genome-wide localisation database Tryptag has provided a comprehensive list of proteins with pore localisation (Billington *et al*, 2023). We took advantage of all these available data to create a revised map of the pore, by combining expansion microscopy with a novel way to globally analyse proximity labelling data. Mostly, our map is in agreement with the earlier map. We did one major correction, which is the placement of the NUP76 complex and the NUP64/NUP98 complex to the cytoplasmic and nuclear site, respectively. The previous symmetric assignment of these complexes was solely based on electron-microscopy detection of immunogold labelled NUP-GFP fusions. This method may be error prone due to large distance of the gold particle to the target protein (GFP+antibody tandem tag) and, even more importantly, it is very difficult to accurately determine the centre of the pore, as membranes are poorly visible. In contrast, in expansion microscopy, the localisation is determined in relation to another pore marker protein and does not depend on membrane detection, and, at least in UExM, the localisation error caused by the antibodies and tag is much smaller because the labelling is done after the expansion. We do therefore believe that expansion microscopy reflects the localisation of pore proteins more accurately. Nevertheless, the conflicting results prompted us to confirm our findings with orthogonal methods and we chose a heat map, generated from proximity labelling mass spectrometry data of marker proteins at different positions within the pore. The heat map agreed with all expansion microscopy data, and, importantly, it does not rely on the proteins being modified with a tag, which could affect localisation. Importantly, both the heat map and the expansion microscopy have their pitfalls and can potentially create wrong results. However, the combination of the methods increases the confidence, in particular when further combined by targeting multiple subunits of one complex rather than just one, as we did for example for the NUP76 complex.”

There is a consistent problem with indicating when proteins are mutants (ie. when tagged with an epitope tag or proximity labelling tag):
Proteins should be referred to in the text and figures with clear indication of the tag and tagged terminus, eg. <protein>::GFP and GFP::<protein> for C and N terminal tagging respectively, whenever data is being derived from a mutant protein. Some figures make this relatively clear, for example Figure 3, while others, for example Figure 1, and the text generally do not.

Tagging terminus and selection of tag can have important biological consequences: Protein tagging may cause an abnormal localisaiton, and it is important that conclusions drawn clearly indicate the evidence - localisation based on a small epitope tag may, for instance, be viewed as more trustworthy than a larger globular proximity labelling protein. Ideally, conclusions should be stated using this information - eg. based labelling by X::TurboID, Y is a ...
For proximity labelling experiments, lack of clear indication of which protein has been TurboID tagged leads to the text being confusing about what is doing the labelling and what is being labelled / which is bait and which is prey (see also below).

Thanks, we have changed the labelling of all fusion proteins throughout in manuscript and Figures accordingly.

A key conclusion is that NUP76 is specific to the cytoplasmic side, there is evidence from HA-tagged NUP76 relative to TY tagged NUP110, and TurboID-tagged NUP76 relative to HA tagged NUP64. However, which terminus of NUP76 was tagged in these instances and, if it was the same one, could that have perturbed its localisation to be on the cytoplasmic side? This affects subsequent interpretation of the prey labelled by TurboID-tagged NUP76. My balanced opinion is that it is unlikely that the tagging will have substantially affected NUP76 localisation, but the mutants proteins used to make the conclusions should be more clearly stated.

We agree, the localisation of the NUP76 complex is a key finding of this paper. Therefore, this conclusion is based on evidence derived from a wide variety of approaches. The main evidence is indeed based on tagged proteins, which can affect the localisation. However: we have imaged not only NUP76, but also NUP140 and NUP149 and all three proteins gave the same localisation. We have tagged all three proteins with HA and imaged with two different expansion methods. In addition, we used streptavidin imaging with one expansion method. Thus, the exclusive cytoplasmic localisation of the NUP76 complex is based on three proteins, each imaged with two different tags and two different expansion methods, which we believe collectively delivers very strong evidence.

Additionally, we have also strong evidence for exclusive cytoplasmic localisation of the NUP76 complex that is entirely tag-independent, and this comes from it being not labelled by any nuclear-site localised components TREX-2 or basket proteins. To further strengthen this, we have now also included TurboID mass spectrometry results from our newly identified nuclear-outer ring marker NUP98 and NUP75 to Figure 2 (A): there is no labelling of NUP76/NUP140 and NUP149. We have also changed Figure 2 (and 3,4) to include all baits to the proximity map, to make this point clearer. And we have changed the text at the end of chapter 2 to:

“In summary, the proximity map accurately predicts the localisations for the vast majority of NUPs. Importantly, it offers orthogonal (tag-independent) validation of the asymmetric localisation of NUP98 and NUP64 to the nuclear site of the pore and of the NUP76 complex proteins to the cytoplasmic site of the pore, confirming the data from expansion microscopy.”

I noted one key concern about interpretation of the data:
There is an important caveat that expansion microscopy (indeed, any microscopy) identifies the location of the small globular fluorescent/TurboID or epitope tag - not the location of the tagged protein.
At the scales being considered, proteins could have a large physical size such that the selected tagged terminus may substantially affect the observed localisation - even in the absence of functional perturbation by presence of the tag.
The proximity data mostly (but not always) uses both N and C terminal fusion with TurboID. Was the motivation here to understand the potentially different location of the N and C terminus? Why not a similar level of care for the microscopy?
When comparing to the existing immuno EM data, carefully and critically consider whether antibodies were directly to the target nuclear pore protein, or if they were to an epitope or fluorescent protein tag and, if so, at which terminus the tag was added.

For the mass spetrometry, we used N and C terminal tagging, for the reason, the reviewer mentioned. For microscopy, we have not used both-end tagging, as it would have massively amplified the amount of expansion experiments which are time-consuming. Also, not all proteins can be tagged on both ends. Instead, we have always used a range of different orthogonal methods to be really sure about the localisation. (see also answer above).

We fully agree with the reviewer that we image the position of the tag, not of the protein, and, for larger tags, this can be an issue. One way to reduce this error is to use post-expansion labelling (UExM) instead of pre-expansion labelling, and this is why we fully switched to UExM after one initital pre-expansion labelling experiment.

We have now added the information about which end the protein tagged and with which tag throughout the paper, for more transparency. We have also added a paragraph to the discussion, to compare our expansion data with the EM-Immunogold data, where we also compare the exact labelling methods (see also answer above).

Problematic terminology.
Please be critical in selecting terms, I found several confusing:
In a paper extensively using microscopy, avoid using the term "focused" outside of referring to the focal plane of a microscope. For example line 320 it is unclear whether you are focusing the microscope or your attention.

We now avoid the word “focused” throuhout the paper.

Avoid the term "nuclear" unless absolutely necessary, given this is a paper about nuclei. For example line 363, I'm left very confused about what "nuclear site" means in this context.

We find it difficult, as the entire paper maps proteins to either site of the pore, and the obvious naming is the nuclear and the cytoplasmic site of the pore. We couldn’t really think of a clearer expression. However, we have now changed “nuclear site” to “nuclear site of the pore” in many places, to make this clearer.

Saying a protein localises to a "single dot" is very unclear when talking about nuclear pores. For example line 450, RanGAP does not resolve as a single dot - in Figure 3C there are ~21 dots, plus a nucleolar signal. Please carefully check and rephrase all of these statements.

We have rephrased all these statements and now use the plural, for example:

“RanGAP and RanBP1 resolved as single dots, well distanced from the NUP64 dots towards the cytoplasmic site, while the RanBPL signals overlapped with the NUP64 signals at the nuclear site, in full agreement with the proximity map. The biotinylation signal of the two proteins with suspected shuttling activity, Ran and MEX67, resolved as large cytoplasmic dots and smaller nuclear dots, connected by a string-like signal reaching through the pore.”

"bait" is commonly used in the proximity labelling literature but, without clear introduction, it is confusing. Especially as "bait" is only meaningful when paired with the "prey" term, which appears not to be used at all. In general, the language here is imprecise, for example 399, bait proteins to not do the labeling, TurboID does the labelling.

As the reviewer says, bait is commonly used in proximity labelling and pulldown experiments, and it is very intuitive to understand. We prefer to maintain this phrase, and we assume that the reader knows that it’s the TurboID that does the labelling.

I realise nuclear pore "spokes" is a term used in the literature, but personally I find this confusing as radial spokes are rings, not spoked disks.

We agree with the reviewer, that this may not be the best terminology, but this is the term that is used in the leading literature of the field, and we feel, it is not up to us to decide to rename it.

Consistently use N and C or amino and carboxy for tagging, mixed terminology is confusing.

Thank you, we now use N and C consistently.

Avoid qualitative terms like "many", and state the exact number, preferably as a fraction of the total (eg. in the abstract "many further asymmetrically localised nuclear pore components" -> "X out of Y nuclear pore proteins, Z of which were novel, were asymmetrically localised")
Throughout the text, we are very specific with numbers and the Figures are self-explaining in this regard. However, in the abstract we do have a strict word limit and its very difficult to be precise, when using numbers. For example, it would require that we set a threshold for what means asymmetric from our mass spectrometry data. Such a threshold would be very arbitrary and we would rather avoid this.

Specific minor points:
Line 29. Please be clear about radial vs. inside-outside symmetry, as written, this is ambiguous.

We could change it to inside-outside symmetry, but, this would somehow implicate that there is no radial symmetry, which we have not investigated, but, it would be rather surprising if its not. To avoid this confusion, we rather stick to the simple word symmetric.

Line 30. Expansion microscopy and TurboID are well-established tools, I'm unclear what is novel here.

We have not seen the combination of expansion microscopy, proximity labelling and streptavidin imaging in any other publication, thus, we believe that this combination is novel, even though the individual methods are not.

Line 38. This is presumably _predicted_ structural homology? Throughout the paper, make it clear when structures are predicted vs experimentally known.

Thank you, we fully agree, that it is important to distinguish and we have changed this in line 38. We also checked the remaining text, but could not find any further inaccuracies.

Line 44. Typo for -> fort

Corrected, thanks

Line 59. Unclear if Nup84/NUP107 means alternative names, or the names for the yeast and human protein respectively. Similar issues arise later.

We have corrected this throughout.

Line 99. This introduction paragraph would benefit from a clear closing question that is addressed by your study.

We prefer to leave this first paragraph as it is, a description and summary of the literature. It is followed by the second paragraph, that indicates the problems and unknowns, and becomes more and more specific to trypanosomes, and our introduction finishes with the research question that we address.

Line 102. "A range or organisms" and "examples" is very uninformative here, as a key impact of your study is the ability to look at the specific evolution of the nuclear pore. A clearer summary of which species, perhaps specifically listing those outside of opisthokonts, would be more informative.

We agree, and we have now included the organisms:

“Structure and composition of nuclear pore complexes has been characterised in a range of organisms, including *S. cerevisiae* (Kim *et al*, 2018; Allegretti *et al*, 2020), human (Kosinski *et al*, 2016), the thermophilic fungus *Chaetomium thermophilum* (Lin *et al*, 2016) and the algae *Chlamydomonas reinhardtii* (Mosalaganti *et al*, 2018).”

Line 109. "Puzzle-stone" isn't a common idiom, at least not one I know.

We now use “stepping stone”.

Line 169. Good practice to indicate the data version, or an indication of when sequences were gathered.

We added the time-scale (2021-2024).

Line 247. Should indicate that this is PBS supplemented with Tween, BSA.

Corrected, thank you

Line 280. This contains some jargon I am not familiar with at all, specifically "reverse and hits only identified by site" should be explained properly.

We have changed to

“Briefly, known contaminants, reverse hits (decoy sequences for calculating the false discovery rate (FDR) and hits only identified by a modification site were filtered out.”

Line 306. Streptavidin imaging is not detection of only autobiotinylation, you also detect all proximal biotinylation, and expect the TurboID to be at the centre of that cloud.

Thanks, we have corrected this:

“followed by the detection of the biotinylation of the bait and proximal proteins with fluorescent streptavidin (=streptavidin imaging)”

Lines 209 to 316. This is not results text, should be discussion(?), or explained with the relevant data.

We assume the reviewer means line 309-316. We agree, that the position to explain this is a little unusual, and we had actually tried a few different versions. However, in the end we felt that putting this information to the beginning of Results is the best solution: we here explain our method and the rationale behind this, which is important in all the chapters of the result section. Streptavidin imaging (and the advantage this has in detecting difficult proteins) is not a commonly known method (we only published this very recently) and we want to familiarize the reader with this approach from the beginning (discussion would be too late).

Line 325. Use consistent terminology for N and C vs. amino and carboxy.

We now use N and C term consistently.

Line 327. The image does not show a single dot, this is unclear. Need to explain the observation more accurately - ie. well-separated points around the periphery of the nucleus in the focal plane.

We have now changed this to “All four proteins resolved as single dots located at the nuclear periphery.”

Line 332. Perhaps I missed it, but is the expansion factor experimentally determined or are you taking this as an assumption into the distance measurement calculation? Needs to be explained/acknowledged as a limitation.

We have now made this clearer: “We experimentally determined the expansion factor as 3.6 and confirmed the isotropic expansion of the nucleus (Figure S1).”

Line 363. "nuclear site" is confusing terminology.

We are now more specific and changed this to "nuclear site of the pore".

Line 368. This is incorrect, you do not resolve the rings at all. There are paired points, around the periphery of the nucleus, which can be interpreted as the inner and outer ring. At no point do you resolve the ring per se. Needs to be explained more clearly.
We changed to "Having confirmed that UExpM has the resolution to distinguish proteins located at the cytoplasmic outer ring from proteins located at the nuclear-site outer ring ..."

Line 388. "bona fide" is not a rigorous term. State the actual scientific logic, are these orthologs of NUPs from other species? Are they NUPs known from prior affinity purification work? etc.

We changed to "Of all the proteins that were labelled by these baits, we initially concentrated on proteins that were previously identified as NUPs, based on predicted structural similarities (DeGrasse *et al*, 2009) and/or affinity purification (Obado *et al*, 2016)"

Line 392. Alphafold does not predict structure or unstructure, it predicts structure with a measure of the accuracy of the predicted structure. Various studies have shown that low structure confidence correlates with regions predicted as unstructured by other dedicated unstructured domain prediction tools. This should be explained more clearly.

We have changed to "For each NUP, we included the pLLDT plots (Wheeler, 2021) to indicate confidence of the predicted structure, which in most cases correlates to structured (high confidence) and unstructured (low confidence, mostly FG-repeats) regions.

Line 395. "that we had placed asymmetrically localised to the nuclear site" is confusing. You haven't placed anything into this system, so why use the word 'placed'? Do you mean you found it localised to somewhere? What does 'nuclear site' mean? Presumably not the nucleus, do you mean the centre of the nuclear pore? Please try to rephrase this to clearer and simpler terms.

We have changed to: "...NUP64/NUP98 that we had localised exclusively to the nuclear site of the pore by expansion microscopy"

Line 405. There are a few places where strong terms like "unequivocally" are used, but

you can rarely be that confident. For example, here, the unequivocal evidence is presumably contingent on TurboID-labelled NUP110 and NUP96 not having their sub-nuclear pore localisation perturbed by the TurboID tag?
We have removed the word “unequivocally”. In fact, the text has changed now, and we use the word “confidently assigned”.

Line 438. Use a term like "concentrated", not "focused".

We now use “concentrated on”.

Line 450. Same "single dot" problem as above. The images show ~20 dots.

We now use the plural.

Line 454. Is the signal a string or a bone? Use a single clear term.

It’s a string that connect the dots, but the whole strucutre (2 dots and a string) is a bone. We believe we have phrased this correctly.

Line 463. "due to phase separation" is not a valid reason here. One model of nucleolar formation is phase separation, but that's certainly not the mechanism maintaining the nucleolus structure in a fixed cell...

We removed “due to phase separation”.

Line 493. This title is not accurate. You are taking known nuclear pore proteins (all based on localisation data from the TrypTag database, I believe?), and finding their likely position within the nuclear pore.

We have changed to

“Predicting the position of unknown proteins within the pore”

Line 604. You have not "assigned" proteins to a localisation, you have determined or discovered that they are on the cytoplasmic side.

We changed to “We detected the NUP76 complex (NUP76, NUP140, NUP149) exclusively at the cytoplasmic site (Figure 1A, E)…”

Line 657. What is the evidence for this? How do you know that this is a "likely" cause?
We changed “likely” to “possibly”.

Line 676. RabGAP in which species? What is the phylogeny of this family, and does that inform the evolution of the family?

For our paper, we feel that this is not really relevant and a detailed description of RabGAP and RanGAP phylogeny would interrupt the flow too much. We do however cite an extensive study about the evolution of RabGAP and RanGAP, so, the reader will find all the information there.

Line 738. A balanced discussion of why previous immuno-EM data gave a symmetrical nuclear pore result is missing.

We have now included a discussion on the discrepancy between the immuno-EM and expansion microscopy data, see above.

Line 739. The conclusion section has strong statements which need more balanced handling, particularly as it seems to be totally separated from discussion. For example this sentence describes the NUP76 cytoplasmic side result as "confidently" and "exclusively", when the actual result is that a tagged (ie. mutant) version of the protein is localised on that side.

We believe that we base this conclusion on a sufficient amount of datasets, including tag-independent approaches, to be "confident" that the NUP76 complex is "exclusively" at the nuclear site (see also answer to some points above). Its not just based on tagged versions of NUP76.

Line 747. Significant should probably be reserved for statistical significance, again this is a sentence replete with strong statements without balanced discussion.

We now use the word "major" instead of "significant".

Line 758. This statement is not true. For example, a fenestrated nuclear envelope, without strong directed transport, may have evolved first - however no extant eukaryotes have been identified which retain this hypothetical ancestral state.
This is a good point, we agree and we have removed this sentence.

Line 760. This was a headline feature of the study, but you draw no specific conclusions.
We touch nuclear pore evolution in the discussion but feel that a further expansion of the topic is beyond the scope of this article.

Figure 1A. Indicate the protein being detected and the tagged terminus, ie. NUP76::HA and NUP110::TY (but correctly indicating N or C-terminal tagging, which is not clear). We have done this throughout the paper.

Figure 1B. Accuracy of this plot is dependent on accurate expansion factor measurement, was that measured? How accurate is your expansion factor? These measurements for the tagged copies of the NUPs, and actual detected proteins (NUP76::HA, etc.) should be in the y axis labels.

We have removed Figure 1B (see explanation above).

Figure 1C-E. As for Figure 1A.

We have done this.

Figure 2A. Question marks in a figure are odd. Either you do or do not have evidence for these statements, please decide if that is the claim you are making. Also unclear if the small subtexts are intended to label that set of proteins or just one.

We removed the question marks and text and used **bold** instead.

Figure 2B,C. The gene name is not "NUP75C", please use proper gene fusion names, ie. NUP75::TurboID and TurboID::NUP158, or otherwise label it clearly. Note that it is important and accurate to show that the prey proteins on the Y axis in A are the untagged wild-type versions.

We have modified Figure 2 to make baits and tag positions clearer.

Figure 1D. Worth noting in the corresponding text that you identified proteins identified as both cytoplasmic and nuclear side-specific, which is a good internal control against eg. biotin accessibility to the nuclear side.
Figure 2B,C. Gene fusion labelling is better, but could be a bit clearer.
We have labelled the fusion proteins clearer.

Figure 4A. Why no clustering/dendrogram analysis, as in Figure 1A?
We have now done this, and also included more proteins for increased accuracy.

Figure 6B. Should show the data points.

Done.

Reviewer #2:

The manuscript "A combination of expansion microscopy and proximity labelling reveals conserved and unique asymmetric functional hubs at the trypanosome nuclear pore" by Gabiatti et al. examine the organization of various nucleoporins within the nuclear pore complex of trypanosomes. Using expansion microscopy and mass spectrometry, the authors redefine the symmetry of previously identified nucleoporins, such as the Nup76 complex, within the structure of the trypanosome nuclear pore complex - the show that a fraction of nucleoporins are asymmetric localized within NPCs as in other organisms and congruent with their assumed role in directed nuclear transport events through the NPC. They also explore the nucleocytoplasmic transport machinery associated with NPCs, focusing on the localization of different transport factors involved specifically in mRNA transport. The proximity based mass spectrometric analysis uncovered several previously unidentified proteins that may play
significant roles in nucleocytoplasmic transport and warrant further investigation.

Overall, I find this study very interesting and technically of very high standard. It provides a huge quantity of relevant data and will significantly advances our knowledge about NPCs in the trypanosome, important also as an organisms outside the ophistokonts for insightful evolutionary comparisons. Nevertheless, I see some minor points regarding the robustness of its conclusions and the extent to which the findings can be confidently interpreted based on the experimental methods employed, which I hope the authors can easily address.

Specific points:

1.) Line 325-326 (also materials and methods) "Upon dual labelling with anti-Ty1 and anti-HA, we carried out expansion and imaging": Why do the authors do the labeling before the expansion? It was described in literature by others that the antibody labeling is done following the expansion. Does this make any difference?

There are two different expansion methods, antibody labelling can be done before expansion (ProExM) or after expansion (Ultra-Expansion-Microscopy). The latter has the advantage of a better resolution (lower linkage error). We are using ProExM only in Figure 1A, all else is Ultra-Expansion-Microscopy.

2.) Line 327-329 "The signals from the NUP76-complex proteins were in all cases clearly separated from the NUP110 signal towards the cytoplasmic site of the pore (Figure 1A and Figure S2 in supplementary material).": The data indeed show that

Nup76, Nup140 and Nup149 are separated from Nup110. Why did the author chose Nup110 instead of a more central localized nucleoporin such as Nup64 (used in figure 1E)? As from figure 1B, it is difficult to get a feeling of how close/far are Nup76, Nup140 and Nup149 from each other which might be due to relating those measurements to the distant Nup110 instead of a more central Nup.

Also in response to reviewer 1, we have now removed Figure 1B, as it gives the wrong impression of an accuracy, that we simply do not have with our method. While we can reliably say, which protein is on which site, its hard to measure the distance: it will depend on which end the tag is and there is also a minor variance in expansion factor between samples.

3.) Line 329-331: "Notably, we observed for every dot signal originating from the NUP76 complex a corresponding NUP110 dot, indicating that trypanosomes, unlike yeast (Galy et al, 2004), do not have basket-less pores.": Why this is not the case for Nup140 and Nup149? Does this represents variabilities in the NPC or is it a result of inefficient labeling?

We do observe this for all three proteins. The NUP76 complex consists of NUP76, NUP140 and NUP149 (explained in line 320).

4.) Figure 2. A proximity map of the trypanosomatid nuclear pore: It is inetersting that Nup76 is seemingly absent at the nuclear ring side. Did the authors try to use other members of the Nup76 complex such as Nup140 and Nup149 as a bait? There seems to be a decent signal for Nup149 in proximity with C-terminal turboID-labeled Nup110.

Yes, NUP149 is indeed labelled a little bit by N-terminally tagged NUP110, but the labelling by NUP76 is much stronger. The heat map is not perfect, for example all the outer ring proteins also label the inner ring. We didn’t do NUP140 or NUP149 TurboID labelling, as, from our various expansion experiments, we were very confident that these are at the cytoplasmic site too. We have now included a paragraph to the discussion, addressing pros and cons and accuracy of our heat map, and, in particular, we stress that the combination of expansion and the heat map increases the confidence in the data.

5.) Line 457-459: "For Ran, Mex67 and RanBPL, we observed an additional signal at the nucleolus, which is defined by the reduction in DAPI stain (Figure S6A), and a minor signal in the nucleoplasm.": Why is it that this bone like signal happens at some NPCs but not others. The RAN images in figure 3C do not align well to the results for RAN in figure 3A where there is no signal detected at all at the nuclear ring side.

Yes, Ran does not label NUP110 and vice versa. However, Ran does label a bunch of nuclear-site proteins, such as RanBPL, NUP64, NUP98 and the lamin NUP2. And, Ran is labelled by NUP98 (ruling out effects of the tag) , which we added to the proximity map in revision. Thus, we are confident that Ran shuttles. We clearly see the shuttling in exansion microscopy too. The fact that we see it in some NPCs but not other is likely technical: in 2D we can only show a single plane, and also, the signal is rather weak.

6.) Line 494-496: "To predict the localisation of the remaining 38 nuclear pore-localised proteins more accurately, we extended the NUP76/96/110 map by the proximity labelling data of Mex67 and Ran and of the outer ring protein NUP158 (Moreira et al, 2023).": Given that Nup110, Nup96 and Nup76 proximity labeling have failed to fully predict RAN and Mex67 binding. Can this analysis here be trusted to reveal the true localization of those 38 proteins? Given that many of them are shuttling nuclear transporter receptors/factors that will probably behave in a similar way to Mex67 and RAN. This might also explain why some unknown proteins such as Tb927.11.1000, Tb927.10.12200, Tb927 and Tb927.10.8160. Can expansion microscopy be used here to reveal the localization some of those unknown proteins?

We fully agree with the reviewer, that the heat map is a prediction, not a proof. We discuss this now in in the first paragraph of the discussion. We found many proteins predicted correctly in this study, but for example shuttling proteins like RAN and MEX67 suffer from poor labelling. We agree that for proof, the proteins would need to be localised, for example by expansion microscopy. This was beyond the scope of this paper. We did expansion microscopy for the three TREX-2 candidates, and all three were at the expected nuclear site of the pore. However, we believe that these data are better off in a follow up paper with some functional data on the TREX-2 complex.

To increase the accuracy and resolution of the map, we have now included NUP98 as an additional bait and include also NUP159 and NUP75 to the major heat map tree.

7.) Line 655-660: "Of the (putative) NUP76 associated proteins, only the pore localisation of NUP140 was clearly abrogated upon NUP76 depletion, while NUP149 and Gle2 still localised to the pore. Note that a slightly diminished pore localisation was observed for all four proteins, likely caused by the disrupted mRNA export and general loss in fitness rather than a specific impact on nuclear pore architecture. Thus, NUP140 localisation to the pore is fully dependent on NUP76, while NUP149 and Gle2 appear to be anchored independent of NUP76.": This is an very interesting finding as in mammals a Nup88 knockdown abolishes Nup214 NPC localization but would not have the same extensive effect on Nup62, which also depends on other nucleoporins for NPC localization.

Thank you! This is nice and we have included this to the discussion:

“Even though the NUP76 complex in trypanosomes is different to the complexes from yeast and human, one similarity is worth mentioning: NUP76 depletion disrupts pore localisation of NUP140, but not of NUP149, just like NUP88 depletion in human disrupts pore localisation of NUP214, but not Nup62 (Bernad *et al*, 2004).”

8.) Figure S2B: the labeling pattern (outside versus inside) seems to be reverted in the upper as compared to the two other panels. Can the authors comment on this?
Thank you! This was a mistake in the image processing (channels were swapped), and we have corrected it.

Reviewer #3:

The authors use a combination of state of the art techniques to better characterize the nuclear pore in Trypanosoma brucei. The apply different expansion microscopy techniques to demonstrate that different than previously thought the nuclear pore of T. brucei actually shows some asymmetries that help to understand the mRNA transport directionality in this system. Furthermore they apply proximity labelling together with mass spectrometry to identify novel components of the pore and verify subcomplex composition of this very important and eukaryote defining structure of the nuclear pore.

From my perspective the major contributions that come from this manuscript are:

1. Establishing the asymmetry of the pore also in Trypanosomes
2. Identification of novel components and verification of subcomplex compositions

The weaker part of the manuscript concerns the functional role of NUP67. The experiments do show that the protein is involved in mRNA export but the statement that it is the functional orthologue of NUP82/88 is in my opinion an overstatement and also not necessary. Using the degron system is nice but I am not sure why RNAi would not also have worked here?

Both RNAi and the degron system would have worked here, the degron system has the advantage that protein degradation is faster and secondary effects are less likely.

We have softened our statement on NUP76 being the functional orthologue to NUP82/88 (“suggesting” instead of “strongly suggesting”), but we would still like to stick

to the idea. In the abstract: “NUP76 holds an essential role in mRNA export consistent with possible functional orthology to NUP82/88.”

The manuscript is overall ok written. I am not a big fan of combining results and discussion this is especially problematic if the manuscript contains much bioinformatics data where there is notoriously a fine line between results and discussion.

I strongly suggest to restructure the manuscript such that the results and discussion are separated.

We have done this, also in response to reviewer 1.

Also many of the figure legends contain results statements. They should be removed.
We have removed results from figure legends, thank you!

This aside I congratulate the authors to a great piece of work that will spike interest in a larger audience and should be published.

Thank you, we are glad you liked it!

Minor comments:

The authors write: Instead, nuclear export is likely fuelled by the GTP/GDP gradient created by the Ran GTPase. The authors speak of the Ran GTPase? is there only one in the T. brucei genome? please be specific here.

There is indeed only one, and we express this by using “the” instead of “a”. We would rather not put more detail to the abstract to not distract from the main topic, as the presence of a single Ran isoform is not so unusual in single cellular organisms.

This is a rather convoluted sentence: The resulting availability of reference proteins for basket, inner ring and cytoplasmic site allowed mapping of all 75 trypanosome proteins with known nuclear pore localisation to a sub-region of the pore based on mass spectrometry data from proximity labelling. What exactly do the authors mean here?
We made it an easier sentence:

"Having defined markers for both sites of the pore, we set out to map all 75 trypanosome proteins with known nuclear pore localisation to a sub-region of the pore using mass spectrometry data from proximity labelling."

Again the following sentence is not really clear: This approach defined many further asymmetrically localised nuclear pore components. I assume they mean to define additional proteins that are asymmetrically localized at the nuclear pore?

We have changed the text to:

"This approach defined several further proteins with a specific localisation to the nuclear site of the pore, including proteins with predicted structural homology to TREX-2 components."

Confirmation of absence is quite a challenging task. I would tone down the statement that you confirmed the absence of Dbp5.

We have removed this statement from the abstract.

The authors should inform that they do not know if the proteins that they have tagged are actually functional and thus might have changed localization it is important to mention this.

We agree that tags can have effects. Therefore, we have been very careful to use several different approaches for our key findings. Our proximity map for example confirms our expansion data in a tag-independent way. We have now made this clearer:

"In summary, the proximity map accurately predicts the localisations for the vast majority of NUPs. Importantly, it offers orthogonal (tag-independent) validation of the asymmetric localisation of NUP98 and NUP64 to the nuclear site of the pore and of the NUP76 complex proteins to the cytoplasmic site of the pore, confirming the data from expansion microscopy."

We have also added a chapter to the beginning of the discussion, critically discussing the robustness of our data.

The authors state that they express from the endogenous locus and thus do not change expression levels. Key for this is the presence 3 prime UTR of the tagged gene, was this maintained? If not please clarify.

Thank you, the 3´UTR is maintained with N-terminal tagging, not with C-terminal tagging, and we used both. Therefore, the reviewer is correct, and we do strictly not in all cases maintain expression levels. We have changed the statement to:

“All fusion proteins were expressed from the endogenous loci, to avoid major changes in gene expression.”

The following statement is too strong and not supported by the data: This phenotype is similar to the one observed upon Nup82 depletion in yeast (Hurwitz & Blobel, 1995; Grandi et al, 1995), strongly suggesting that NUP76 is the functional orthologue to yeast Nup82 with a crucial role in mRNA export. I am sure the depletion of many components of the nuclear pore will lead to a lack of mRNA export. I think the authors need include further support for their claim.

We have removed “strongly”, and weakened this statement also in the abstract, also in response to reviewer 2. However, additional evidence comes from us (Proximity labelling data) but also from high stringency pulldown data of others:

“Under high stringency conditions, we found TbMex67 co-isolated with TbNup76, TbNup140, and TbNup149, as well as the highly conserved binding partner of Mex67, TbMtr2. This strongly implies that the TbNup76 complex is part of the mRNA export factor docking platform.”.

Obado, S. O. *et al.* Interactome Mapping Reveals the Evolutionary History of the Nuclear Pore Complex. *PLoS biology* **14**, e1002365 (2016).

We are also discussing this topic:

“The NUP76 complex had been previously suggested to be part of the mRNA remodelling platform, as all three proteins co-isolated with Mex67 under high stringency conditions (Obado *et al*, 2016). Consistent with this hypothesis, we now show exclusive cytoplasmic localisation of the NUP76 complex (Figure 1) as well as significant structural similarities of NUP76 with the mRNA remodellers of yeast and human, NUP82 and NUP88, respectively (Figure 5 and Figure S9) and nuclear mRNA accumulation upon NUP76 depletion (Figure 6C).”

Figure 6:
The authors make a great effort to characterize the organization of the pore by expansion microscopy using the relative positioning of individual components. But in the degron experiment they claim the pore assembly is not affected by simply showing one components, which could be different in its localization upon the depletion of NUP76 affecting the pore function indirectly. In order to make a solid claim here the authors could one or two components they analysed in detail above and show their relative localization does not change. This would be much stronger evidence.

We agree that one component is not enough. We therefore say “suggesting that” the pore stays contact (not proving). In addition, the localisation of Gle2 and NUP149 is not

affected by the NUP76 depletion, and these can serve as two further controls here, and we included this statement to the text:

"Of the (putative) NUP76 associated proteins, only the pore localisation of NUP140 was clearly abrogated upon NUP76 depletion, while NUP149 and Gle2 still localised to the pore (serving as additional controls for pore integrity not being affected)."

The authors state: Notably, we observed for every dot signal originating from the NUP76 complex a corresponding NUP110 dot, indicating that trypanosomes, unlike yeast (Galy et al, 2004), do not have basket-less pores.
The authors could discuss this point? does this mean there are no assembly intermediates?

We would prefer not to go in too much detail here, as this is out of the focus of this paper. Also, it seems more that yeast is the exception rather than trypanosomes, was, to the best of our knowledge, they are the only species with basket less pores.

corrections:
Altogether, the combination of proximity labelling with expansion microscopy revealed an asymmetric architecture of the trypanosome nuclear pore supporting inherent roles fort directed transport. "for" instead of fort.

Thank you, corrected.

...but with a fundamental different mRNA remodelling platform at... fundamentally

Thank you, corrected.
